# Airway microbial communities, smoking and asthma in a general population sample

**DOI:** 10.1016/j.ebiom.2021.103538

**Published:** 2021-08-20

**Authors:** Elena M. Turek, Michael J. Cox, Michael Hunter, Jennie Hui, Phillip James, Saffron A.G. Willis-Owen, Leah Cuthbertson, Alan James, A.William Musk, Miriam F. Moffatt, William O.C.M. Cookson

**Affiliations:** a National Heart and Lung Institute, Centre for Genomic Medicine, Imperial College London SW3 6LY, United Kingdom; b School of Population and Global Health, University of Western Australia, Australia; c Busselton Population Medical Research Institute, Western Australia, Australia; d PathWest Laboratory Medicine, Queen Elizabeth II Medical Centre, Western Australia, Australia; e Department of Pulmonary Physiology, Sir Charles Gairdner Hospital, UWA Medical School, University of Western Australia, Australia; f Department of Respiratory Medicine Sir Charles Gairdner Hospital, UWA Medical School, University of Western Australia, Australia

**Keywords:** Airway microbiome composition population asthma smoking

## Abstract

**Background:**

Normal airway microbial communities play a central role in respiratory health but are poorly characterized. Cigarette smoking is the dominant global environmental influence on lung function, and asthma has become the most prevalent chronic respiratory disease worldwide. Both conditions have major microbial components that are incompletely defined.

**Methods:**

We investigated airway bacterial communities in a general population sample of 529 Australian adults. Posterior oropharyngeal swabs were analyzed by sequencing of the 16S rRNA gene. The microbiota were characterized according to their prevalence, abundance and network memberships.

**Findings:**

The microbiota were similar across the general population, and were strongly organized into co-abundance networks. Smoking was associated with diversity loss, negative effects on abundant taxa, profound alterations to network structure and expansion of *Streptococcus* spp. By contrast, the asthmatic microbiota were selectively affected by an increase in *Neisseria* spp. and by reduced numbers of low abundance but prevalent organisms.

**Interpretation:**

Our study shows that the healthy airway microbiota in this population were contained within a highly structured ecosystem, suggesting balanced relationships between the microbiome and human host factors. The marked abnormalities in smokers may contribute to chronic obstructive pulmonary disease (COPD) and lung cancer. The narrow spectrum of abnormalities in asthmatics encourages investigation of damaging and protective effects of specific bacteria.

**Funding:**

The study was funded by the Asmarley Trust and a Wellcome Joint Senior Investigator Award to WOCC and MFM (WT096964MA and WT097117MA). The Busselton Healthy Ageing Study is supported by the Government of Western Australia (Office of Science, Department of Health) the City of Busselton, and private donations.

**Research in context**

**Evidence before this study**

The airways of the lung carry commensal microbiota in a similar density to the microbiome of the small intestine. The large surface area of the lungs and constant infective challenges mean that the respiratory microbiota have profound opportunities to affect mucosal immunity. However, there is still uncertainty as to what extent bacteria in healthy lungs are part of a resident ecosystem, or whether their presence is transient and driven stochastically by exposure. As yet there is no data as to which human airway organisms underpin mucosal health.

We therefore investigated the ecology and structure of normal airway microbial communities in a general population sample, as a first step to defining organisms associated with health, in contrast to airway microbiota associated with the major respiratory conditions of cigarette smoking and asthma.

**Added value of this study**

We found that the airway organisms were very similar throughout the general population. Strong positive and negative correlations were seen between the abundances of different taxa, revealing networks within the airway communities. Smoking was associated with profound alterations to network structure and expansion of *Streptococcus* spp. By contrast, the asthmatic microbiota were selectively affected by loss of diversity, an increase in *Neisseria* spp. and by reduced numbers of low abundance but prevalent organisms.

**Implications of all the available evidence**

Our study shows that the healthy airway microbiota are contained within a highly structured ecosystem, suggesting balanced relationships between the microbiome and human host factors. The marked abnormalities in smokers may contribute to chronic obstructive pulmonary disease (COPD) and lung cancer. The narrow spectrum of abnormalities in asthmatics encourages investigation of damaging and protective effects of specific bacteria.

Alt-text: Unlabelled box

## 1 Introduction

The airways of the lung carry commensal microbiota that make essential contributions to respiratory health [1,2]. The surface area of the lungs is 40–80 m^2^, compared to 30 m^2^ metres sqaured and not reference 2"?> in the gut [2], and the respiratory microbiota have profound opportunities to affect mucosal immunity. We have therefore investigated the ecology and structure of normal airway microbial communities in the general population, as a first step to defining organisms associated with health as well as major respiratory diseases.

Cigarette smoking and asthma are global conditions with major microbial components that are also incompletely defined. A quarter of men and 5% of women in the world smoke cigarettes daily [3]. Smoking causes 11.5% of deaths globally [3] and chronic obstructive lung disease (COPD) and lung cancer are its most common pulmonary consequences. Smoking has distinctive effects on airway microbial communities [4]. COPD, even in its early stages, is accompanied by recurrent infections [4,5] and airway bacteria may contribute to lung carcinogenesis [6].

Asthma is an inflammatory disorder of the airways that has become the most prevalent chronic respiratory disease worldwide [7,8]. In numerous studies its rise has been linked to urbanization and the loss of traditional rural environments [9], [10], [11]. When applied to the airways, the “hygiene hypothesis” suggests that loss of microbial exposure allows asthma to develop [12,13], but it is not known how loss of bacterial diversity predisposes to asthma. Possibilities include reduced signals from commensal organisms that normally down-regulate mucosal immune responses [14]; and that inflammation follows intermittent mucosal damage by the pathobionts (potentially pathogenic bacteria) that are in excess in asthmatic airways [1,[15], [16], [17], [18].

Manipulation of the bowel microbiota has been successful in treating several conditions [19], and it is reasonable to consider that the airway microbiota might also be modified therapeutically [2]. However, there is uncertainty as to what extent bacteria in healthy lungs are part of a resident ecosystem or whether their presence is transient and driven stochastically by exposure [20]. As yet there is no data as to which airway organisms underpin mucosal health.

We therefore sought to test airway community composition in a general population sample from a cross-sectional community-based prospective cohort study of 'Baby Boomers' (born from 1946 to 1964) living in the Shire of Busselton, in South-Western Australia [21]. Busselton is a coastal city with a warm-summer Mediterranean climate and minimal air pollution. Tourism, services and retail are the primary sources of income. The prevalence of respiratory disease is similar to other Australian centres [22,23].

We compared changes in diversity associated with the strong environmental factor of smoking with more subtle alterations that might influence asthma. We defined bacterial taxa by amplicon sequencing of the 16S ribosomal RNA gene. However, 16S sequences do not differentiate at all well between *Streptococcus* spp. [24] even though they are abundant in the respiratory tract and exhibit high rates of clonal diversity [25]. It has been shown that *Streptococcus* spp. may be much better identified by variation in sequences from selected other genes, including methionine aminopeptidase (*map*) [24]. We therefore sequenced a *map* amplicon to further differentiate between *Streptococcus* taxa [24].

Diseases with microbial components should be considered in the context of the complex ecosystems formed by interactions between the human microbiota themselves and with their host environment. These interactions are fundamental to the beneficial or pathogenic behaviour of individual taxa. Complex patterns of microbial abundance have been reported in the bowel (where they are known as enterotypes) [26], vagina [27], mouth [28], and skin [28], but not yet in the airways.

The methodology for identification and characterization of major patterns in microbiomes is not yet fixed [29]. Network analysis is effective in revealing ecological interactions within microbial communities [30], and so we applied weighted correlation network analyses (WGCNA) [31] to our dataset. The analysis identifies positive and negative correlations in abundance between different bacterial taxa, alternatively suggesting mutual co-operation or inhibition. It also specifies the OTUs that are most connected to others (i.e. that are hubs in the networks), suggesting candidates for the strongest influences on community structure.

Direct sampling of the lung microbiota requires invasive procedures, such as bronchoscopy, that are difficult in epidemiological studies. The nasal microbiome differs significantly from the oropharynx [32], perhaps because nasal environmental exposures are unfiltered and the nasal epithelium differs histologically and functionally from lower airways [33]. Nevertheless, the nose may provide a source for seeding pathogens into the lower airways. The oropharynx and the intra-thoracic airways form a contiguous tract with air, mucus and microbes moved in both directions by respiration and the muco-ciliary ladder [20,34]. The lower airway microbiome is broadly similar to that of the oropharyngeal airway [15,^20^,34], although the abundance of pathogens in the lower airways of diseased subjects is imperfectly reflected in the oropharynx [15,35]. Whilst recognising these limitations, we sampled our population using posterior oropharyngeal swabs taken beyond the tonsils and palate and near to the top of the muco-ciliary ladder.

## 2 Methods

### 2.1 Ethics

The study has received ethics approval from the University of Western Australia Human Research Ethics Committee (Number RA/4/1/2203). All subjects gave written informed consent to participation in the study.

### 2.2 Subject recruitment

Through the Busselton Health Study in Western Australia, we recruited 578 Caucasian adults. These subjects gave a 15% margin above a minimum sample size of 500 subjects (power estimations described below in Statistical analysis) to cover uncertainty about DNA extraction in London from samples collected in Australia. (DNA extraction and downstream analyses subsequently proved to be robust). Individuals with a diagnosis of cancer were excluded, but otherwise no selection was made for subject status. Subjects completed a detailed questionnaire as previously described [21]. Subjects were classified as asthmatic if they answered yes to the question “Has your doctor ever told you that you have asthma”.

Samples for microbial analysis were taken under direct vision, using sterile rayon swabs that were rubbed gently with an even pressure around the posterior oropharynx five times, strictly avoiding contact with tongue, tonsils, palate or nose. Swabs were immediately frozen and stored at −80 °C prior to transportation on dry ice to Imperial College London, UK.

### 2.3 16S rRNA gene sequencing

DNA was extracted from swab heads using the MP Bio FastDNA Spin Kit for Soil (http://www.mpbio.com). A single sample was examined for each subject.

PCR of the 16S rRNA V4 region was performed in quadruplicate using a custom indexed forward primer S-d-Bact-0564-a-S-15 (5′ AYT GGG YDT AAA GNG 3′), reverse primer S-D-Bact-0785-b-A-18 (5′ TAC NVG GGT ATC TAA TCC 3′) and a high fidelity Taq polymerase master mix (Q5, New England Biolabs, Massachusetts, USA). Primer sequences were based on Klindworth et al. [36], with dual-barcoding as per Kozich et al. [37] with adaptors from Illumina (California, USA). A mock community [38] was included to assess sequencing quality. PCR cycling conditions were: 95 °C for 2 min followed by 35 cycles of 95 °C for 20 s, 50 °C for 20 s and 72 °C for 5 min. Amplicons were purified, quantified and equi-molar pooled and the library paired-end sequenced (Illumina MiSeq V2 reagent kit) as previously described [38]. Bacterial load was quantified by qPCR using KAPA BioSystems SYBR Fast qPCR Kit with the same 16S rRNA V4 primers used for sequencing.

Analysis of data was carried out in the R environment and details can be followed on github: https://tinyurl.com/y2onjblt. Sequence processing was performed in QIIME (Version 1.9.0) [39]. Community level differences in alpha and beta diversity and Operational Taxonomic Unit (OTU) level differences, were analyzed using Phyloseq in R (Version 3.2.0). A phylogenetic tree was generated from the representative sequences using the default parameters of the make_phylogeny command [39]. Taxonomy of OTUs was assigned by matching representative sequences against release version 23 August 2013 of the Silva database [40] using the default parameters of the assign_taxonomy command [39]. OTUs occurring in only one sample or with less than 20 reads in the whole dataset were removed. Weighted and unweighted UniFrac beta diversity measures and subsequent principal co-ordinates analysis of them was carried out using the beta_diversity_through_plots script [39]. For the purposes of alpha diversity calculations, the raw counts tables were rarefied to a minimum of 6,543 reads. Significant differences in alpha diversity between datasets were assessed using Mann–Whitney U-tests.

At the time of the initial laboratory study (2012 – 2013) the potential risk of sample contamination from laboratory reagents was not known or fully understood [41]. Potential contaminant OTUs were identified by the presence of negative Spearman's correlations between OTU abundance and bacterial burden (logged qPCR copy number), adjusted using Bonferroni corrected *P*-values < 0.05. OTUs subsequently of interest were cross-checked with a listing of potential contaminants [41].

### 2.4 Map gene sequencing

We further differentiated *Streptococcus* spp. by sequencing the methionine aminopeptidase (*map*) gene [24] in 483 samples (constrained to 5 sequencing runs with controls). Of these subjects 234 were never-smoking, 196 were ex-smokers, and 53 were current smokers. We used barcoded primers map-up 5′ GCWGACTCWTGTTGGGCWTATGC ‘3 and map-down 5′ TTARTAAGTTCYTTCTTCDCCTTG ‘3. As positive controls, DNA from nine strains of *Streptococcus* with bacterial identity confirmed through Sanger sequencing was used for positive controls (S. agalactiae (DSMZ-2134); *S. constellatus* subsp. *Constellatus* (DSMZ-20,575); *S. infantis* (DSMZ-12,492); *S. parasanguinis* (DSMZ-6778); *S. pneumoniae* (DSMZ-20,566); *S. pseudopneumoniae* (DSMZ-18,670); *S. pyogenes* (DSMZ-20,565); *S. sanguinis* (DSMZ-20,567); and *S. mitis* (DSMZ-12,643)). Analysis was performed in QIIME [39], using a clustering level of 95% with closed picking to define OTUs. We attributed the most common *map* gene OTU sequences to Streptococcal species by BLAST searches. Full details are online (http://hdl.handle.net/10044/1/63937).

### 2.5 Statistical analysis

Following the convention that OTU data is similar in distributions and complexity to the results of RNA sequencing, we estimated power to detect differences in OTU abundances with RnaSeqSampleSize [42]. Assuming 250 experimental subjects in each group, prior data indicates that the minimum average read counts among the prognostic OTUs in the control group to approximate 10,000, the maximum dispersion 0.5, and the ratio of the geometric mean of normalization factors to be 1. Assuming the total number of OTUs for testing to be 500, that 50 are prognostic, and the desired minimum fold change is 1.4, we were able to reject the null hypothesis that the population means of the two groups are equal with probability (power) 0.97 using an exact test. The FDR associated with this test was 0.01.

Stepwise multiple and logistic regression models were used respectively to explore microbial diversity and inhaled corticosteroid use (IBM SPSS Statistics Version 25). Missing values were deleted pairwise.

We used the Differential Expression Analysis for Sequence Count Data (DESeq2 function in R) [43] to compare OTU abundance between subject and control groups, controlling the false positive rate at *P* *=* 0.05. Parameters extracted for each OTU included log_2_(fold change), globally adjusted *P* value and abundance and prevalence information. Two-sided *P* values are reported throughout.

Co-abundance networks between non-rarefied OTU abundances were analyzed using the WGCNA package (version 1.51, R version 3.3.2 [2016–10–31]) [44]. Abundances were log transformed with 0.1 added to zeroes [45], and the topological adjacency matrix was constructed from Spearman's correlation coefficients with a *β* soft thresholding parameter of 3. Hierarchical clustering of the overlap matrix with dynamic tree cutting defined the co-abundance modules, with a minimum module size set at 20 OTUs. The MM (module membership) was defined as the correlation of gene expression profile with the module eigengene. The significance of Spearman's correlation between module eigengenes and clinical variables was adjusted for multiple testing using the Benjamini and Hochberg method [46]. Module structure was visualised and contrasted between cohorts using the R package circlize (0.4.5).

### 2.6 Role of funding sources

The Funders had no role in study design, data collection, data analyses, interpretation, or writing of the report.

## 3. Results

### 3.1 Structure of the normal airway microbiome

We submitted oropharyngeal swabs from 578 subjects to 16S rRNA gene qPCR and sequencing, the latter yielding 44,290,100 high quality reads (Supplementary Fig. 1 for analysis structure). After removal of 173 OTUs with high probability of being contaminants and 13,472 rare OTUs present in only one sample or with less than 20 reads, there remained 4218 OTUs derived from 43,775,771 reads. To enable diversity analyses based on proportions, the samples were rarefied to a minimum of 6543 reads, retaining 529 samples containing 4005 OTUs and 3,461,247 reads. For consistency, unrarefied data from these same 529 samples were used to test differences between subject groups by DESeq2, and as a basis for network analyses. No systematic differences in results were seen if the larger sample was analyzed.

Non-respiratory diagnoses potentially influencing the microbiome were diabetes (*n* = 18 patients) and gastro-oesophageal reflux (GERD, *n* = 36). No associations were found for diabetes or GERD in any analyses, and we classified subjects with these diagnoses as unaffected.

The average age of the 529 subjects was 56 years (Supplementary Table 1). Sixty subjects were current smokers and 216 were ex-smokers (with a mean 18 years since quitting). The mean levels of the forced expiratory volume in one second (FEV1) and the forced vital capacity (FVC) of the subjects were within the normal range [47] (Supplementary Table 1). There were 77 doctor-diagnosed asthmatics, 82% of whom were atopic by prick skin tests (47% of the rest of the population were also atopic). Just 27 (35%) of our asthmatics were currently using inhaled corticoid steroids (ICS), indicating a preponderance of mild disease. There was only one case with a clinical diagnosis of COPD, fewer than the 7% anticipated [23]. The frequency of asthma and current smoking were not different to the whole Busselton cohort [21].

Subjects were not included if they were taking antibiotics within six weeks of the time of study. The annual rate of antibiotic prescription in the Australian population is 254 per 1000, and half of these will be for respiratory infections [48], so it is likely that many smokers will have intermittently been given antibiotics. Asthma was not currently considered an indication for antibiotics in the Australian healthcare system.

An estimate of Bray Curtis beta diversity (β) for the population gave the mean dissimilarity in microbial diversity (M) between subjects to be 0.51 ± SD=0.06 (on a scale of 0–1), indicating that on average individual airway microbiomes shared about half of their OTUs. No significant differences in β were observed between disease phenotypes through PERMANOVA (Adonis function in R).

Five phyla contained 98.4% of all OTUs (Table 1, Supplementary Table 2). Firmicutes (predominately *Streptococcus* and *Veilonella* spp.) was the most common phylum, with 24 OTUs in the top 50, and 57.9% of all OTUs found in the complete dataset. Bacteroidetes (predominately *Prevotella* spp.) contained 14.1% of the OTUs, Proteobacteria (predominately *Neisseria* and *Haemophilus* spp.) contained 12.3%, Actinobacterium 9.1% and Fusobacterium 4.9%. Overall, the 50 most abundant OTUs accounted for 92% of the data (Supplementary Table 2).

**Table 1:** Principal phyla and genera of airway bacteria in a general population sample.

Phylum	Genus	Abundance*	Phylum	Genus	Abundance*
*Firmicutes (53.4%)*		*Bacteroidetes (17.7%)*			
	*Streptococcus*	18.92%		*Prevotella*	15.36%
	*Veillonella*	13.74%		*Porphyromonas*	1.45%
	*Unidentified_Firmicutes*	11.79%		*Capnocytophaga*	0.73%
	*Selenomonas*	1.71%		*Tannerella*	0.09%
	*Gemella*	1.64%		*Bergeyella*	0.08%
	*Granulicatella*	1.45%	***Fusobacteria (8.5%)***		
	*Johnsonella*	0.70%		*Fusobacterium*	4.40%
	*Lachnoanaerobaculum*	0.69%		*Leptotrichia*	4.09%
	*Megasphaera*	0.66%	***Proteobacteria (8.5%)***		
	*Not known*	0.46%		*Neisseria*	4.59%
	*Stomatobaculum*	0.43%		*Haemophilus*	3.48%
	*Oribacterium*	0.43%		*Not known*	0.33%
	*Solobacterium*	0.23%		*Campylobacter*	0.07%
	*Peptostreptococcus*	0.17%	***Actinobacteria (7.2%)***		
	*Peptococcus*	0.16%		*Actinomyces*	4.62%
	*Parvimonas*	0.16%		*Atopobium*	2.11%
	*Butyrivibrio*	0.10%		*Rothia*	0.36%
	*Catonella*	0.05%		*Bifidobacterium*	0.08%
	*Filifactor*	0.05%	**Other (0.25%)**		

*Abundance based on total 43,652,299 high-quality sequence reads in 529 subjects.

*Streptococcus* spp. show high rates of clonal diversity and are poorly differentiated by standard culture and 16S sequences [24,25]. We therefore sequenced the methionine aminopeptidase gene (*map*) to further differentiate between *Streptococcus* taxa [24] in 483 subjects. After removal of *map_*OTUs only present in one sample or with fewer than 20 reads or negative correlations with qPCR abundance there remained 14,898 *map_*OTUs (Supplementary Fig. 2), suggesting substantial variation in Streptococcal strains in the population. β diversity estimates in rarefied data (to a level of 7700 reads) found *M* = 0.84 ± SD = 0.06, indicating low similarity of the streptococcal composition between subjects. The nine most prevalent *map_*OTUs were identified as *S. salivarius*, with *S. parasanguinis* the tenth most prevalent. (Supplementary Table 7). The potential pathogen S. *mitis/pneumoniae* was detected in 58% of subjects, although at low abundance.

Microbial communities are formed through complex ecological interactions that can be uncovered through network analyses [30]. On the assumption that correlations in the abundance of different taxa would reflect co-ordinated growth, we applied weighted correlation network analyses (WGCNA) [31] to the Busselton dataset.

We observed 13 discrete modules in which the abundance of members was strongly correlated. Just 13 OTUs remained unassigned to a network. The WGCNA program labels modules with unique colour identifiers, but we have also named them according to their most abundant genera (Table 2). Unassigned OTUs are referred to as the grey module. The 5 largest modules (in terms of abundance of members) contained 97.6% of all OTU sequence reads (Table 2).

**Table 2:** General population microbiome module summary and associations with smoking.

Module ID (Colour)	Number of OTUs	Total Abundance	Overall%	Cum%	Smoking R	Smoking *P*	Module description
Prevotella.1 (Turquoise)	2218	18,636,985	42.69	42.69			Commensal carpet: *Veilonella, Prevotella, Actinomyces. Veillonella* and *Atopobium* hubs
Streptococcus.2 (Blue)	472	9433,313	21.61	64.30	−0.13	2.E-02	*Streptococcus* and *Haemophilus* prevalent. *Lactobacilliae* and *Gemella* hubs
Streptococcus.1 (Magenta)	126	8480,289	19.43	83.73	0.18	8.E-04	*Streptococci* dominated
Fusobacteria (Brown)	583	3099,110	7.10	90.83	−0.26	4.E-08	*Fusobacteria* and *Leptotrichia* hubs
Neisseria (Green)	204	2969,651	6.80	97.63	−0.35	1.E-14	*Neisseria* dominated, prevalent *Capnocytophagia*
Prevotella.2 (Black)	136	387,098	0.89	98.52	0.15	4.E-03	*Prevotella, Parvimonas, Streptococci, Porphryomonas*
Veillonella (Cyan)	50	173,186	0.40	98.92	0.17	1.E-03	*Veillonella*
Prevotella.3 (Purple)	71	105,630	0.24	99.16			*Prevotella* dominated
Indeterminate (Tan)	55	101,284	0.23	99.39	0.15	4.E-03	*Prevotella* and *Treponema*
Porphymonas (Salmon)	50	89,951	0.21	99.60	0.15	6.E-03	*Porphyromonas* and *Prevotella*
Bifidobacteria (Pink)	134	86,562	0.20	99.80	0.32	8.E-13	*Bifidobacterium* hubs
Peptococcus (Midnightblue)	44	79,920	0.18	99.98	−0.16	2.E-03	*Peptococcus*
Contaminants (GreenYellow)	62	8869	0.02	100.00	−0.12	3.E-02	*Herbaspirillum*: potential contaminants
Unconnected (Grey)	13	451	0.00				Unconnected OTUs: potential contaminants

Individual hubs were strongly connected to their network vectors (range of *P* = 7.9E-266, MM (module membership: correlation with the module eigengene) = 0.95 to 1.9E-121, MM = 0.81) (Supplementary Table 4), and the strengths of association suggest a hypothesis that these co-abundance modules may represent “guilds” of co-operating bacteria that occupy ecological niches on the mucosa.

The largest guild (turquoise module: Prevotella.1) accounted for 42.7% of reads (Table 2, Supplementary Table 4). The most common organisms were within the genera *Prevotella, Veillonella, Actinomyces* and *Atopobium*. These organisms resemble common mucosal commensals at other body sites, and perhaps represent a base microbial carpet. The smaller guild (cyan) on the same division (B) of the network dendrogram (Supplementary Fig. 3) was almost entirely made up of *Veillonella* spp. and may occupy a related ecological niche.

The blue module (Streptococcus.2) contained 21.6% of reads, predominately from the genera *Streptococcus, Haemophilus* and *Veillonella*. Network hubs included *Lactobacillales* and *Gemella*. The adjacent network (Neisseria: green) (Supplementary Fig. 3) was dominated by *Neisseria*, with *Porphyromonas*, and *Capnocytophagia*. This may suggest a normal guild than can be occupied by *Proteobacteria* potential pathogens.

The magenta module (Streptococcus.1) (19.4% of reads) was completely dominated by *Streptococcus* taxa (40%) and an unidentified *Firmicutes* (60%) (Supplementary Table 4) which is likely also to be streptococcal (based on phylogenetic clustering, not shown). Network hubs were also *Streptococcus*, identifying a streptococcal-specific guild in the mucosa.

A stepwise multiple regression (IBM SPSS Statistics v25) found that microbial diversity in individual airways was independently related to current cigarette smoking (R^2^ = 6%, *P* < 0.001), a current diagnosis of asthma (additional R^2^ = 1.4%, *P* < 0.005) and packyears of smoking (additional R^2^ = 0.8%, *P* = 0.04) (Supplementary Table 3), but not to age or sex. We therefore partitioned the data into three subgroups: smoking + packyears> 10 (*n* = 159); asthmatic (*n* = 77); and unaffected (*n* = 300). The seven asthmatics who were current smokers were included in both smoking and asthmatic subgroups. Excluding these individuals made no difference to our significant findings (data not shown).

### 3.2 Smoking

**Fig. 1:**
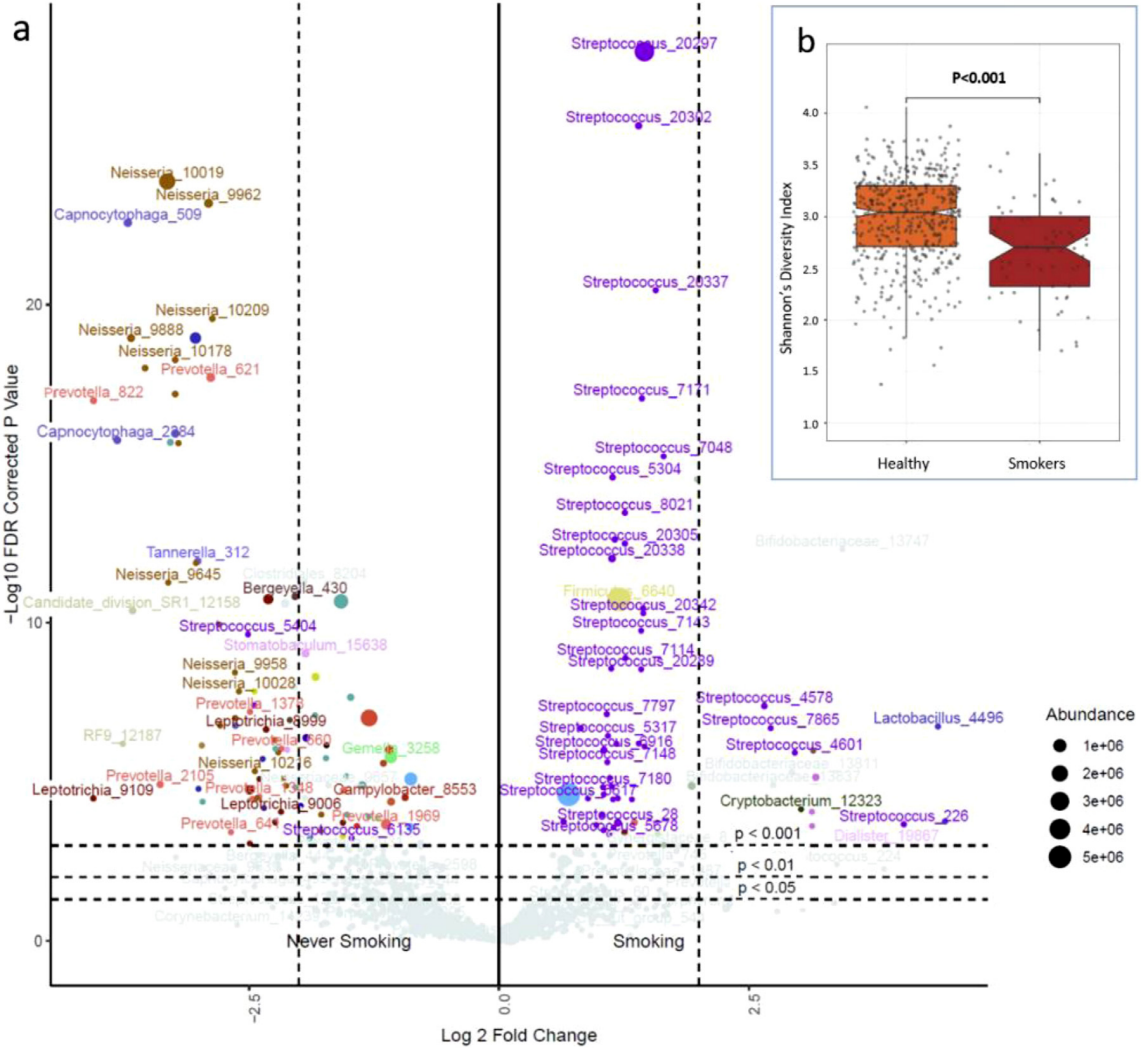
Smoking and the airway microbiome, (a) The volcano plot shows significant differences in the abundance of OTUs between current smokers and the rest of the population. Fold change is shown on the x axis and -log10 *P* (FDR corrected) on the y axis. Relative abundances are reflected in the data point sizes; (b) shows differences in alpha diversity between smokers and never smokers (boxes show inter-quartile range, notches 95% CI of the median, *P* values are two-sided from multiple regression).

A DESeq2 analysis to identify significant differences in the abundance of specific taxa revealed marked effects of cigarette smoking. (Fig. 1, Supplementary Fig. 4, Supplementary Table 5a and b). The loss of diversity affected many abundant OTUs, including those in the genera *Fusobacterium, Neisseria, Haemophilus, Veillonella* and *Gemella*. By contrast, the OTUs increased in smokers were in general highly abundant *Streptococci*. Examination of *map* gene OTUs attributed increases in abundance to *S. parasanguinis* (log_2_(Fold change) 5.2, *P_adjusted_*=1.75E-07), *S. mitis/pneumoniae* (3.62, 4.81E-09), *S. salivarius* (3.03, 5.59E-15) and *S. thermophilus* (2.53, 7.38E-05) (Supplementary Table 8).

To further explore the impact of smoking and asthma on the higher order structure of the airway microbiome, co-abundance networks were constructed separately in the asthmatic and current smoker portions of the cohort and compared with the full dataset (representing the whole population) (Supplementary Fig. 4). We limited direct comparison to the 4207 OTUs present in all datasets. Including the remaining 13 OTUs made no difference to the conclusions.

**Fig. 2:**
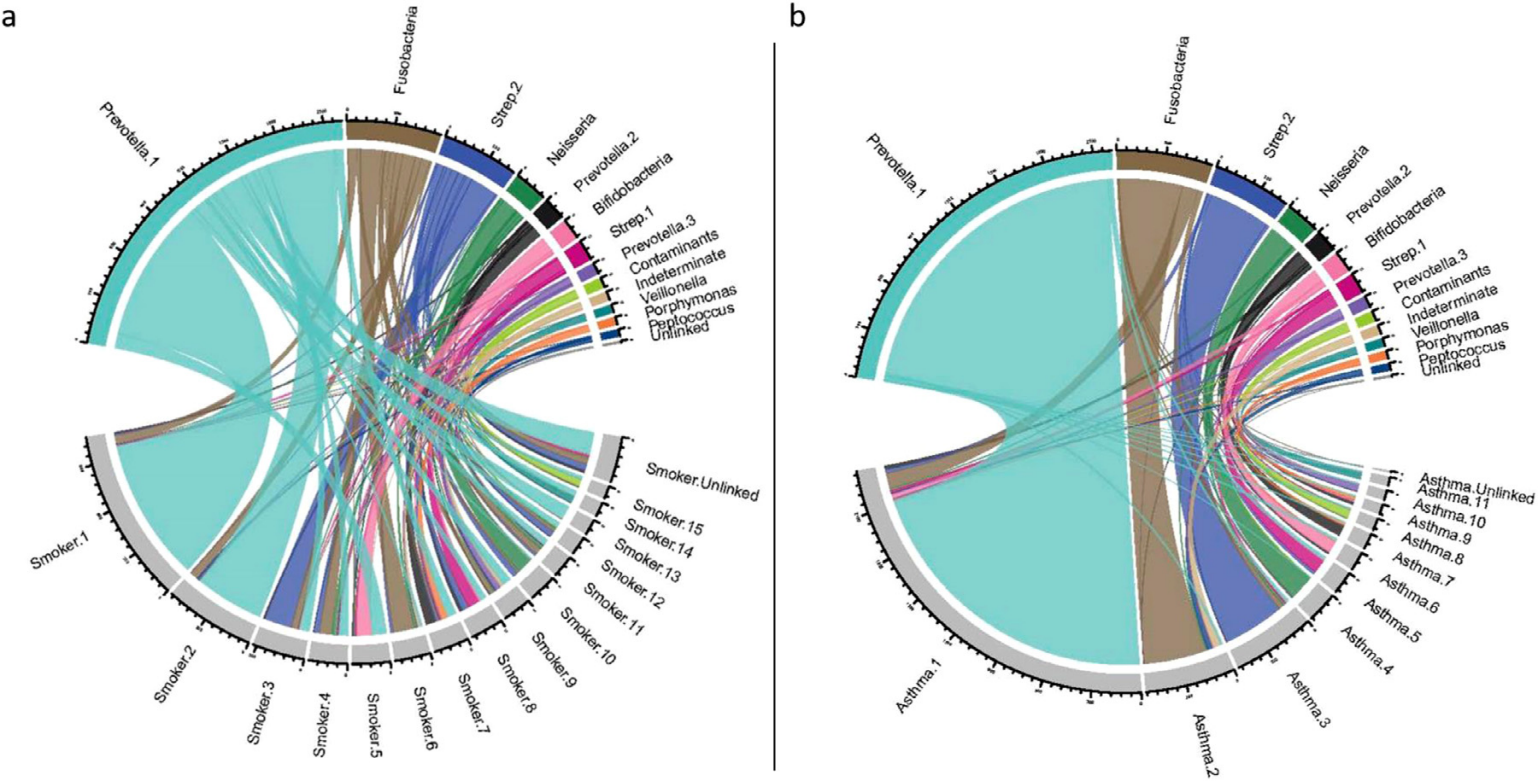
Network structure of the airway microbiome in normal subjects, compared to smokers and asthmatics The Chord plots show sharing and discordance of 4207 OTUs common to the three datasets for co-abundance networks. (a) Network membership in the whole population (top half of plot) compared to current smokers (bottom half of plot); and (b) compared to asthmatics. Module colours are arbitrarily assigned by WGCNA, and module bacterial names are derived from Table 2. Modules in smokers and asthmatics are simply named by size (Smoker.1, Asthma.2, etc.). There is a marked change of structure with fragmentation of major networks in the smokers, but high conservation of network membership between asthmatics and the whole cohort.

The network structure of the communities was profoundly altered in current smokers. Whilst the largest guild (Prevotellla.1: commensal carpet) showed relative preservation, other modules showed markedly lower levels of conservation and were strongly positively or negatively associated with smoking status; either in terms of module eigenvectors or hubs (Fig. 2, Table 2, Supplementary Table 4). In smokers, 276 OTUs were not included in any module, meaning that their abundances were no longer correlated with other organisms. Unconnected taxa most strongly featured *Streptococcus* (70 OTUs), unknown genera (41 OTUs) and *Veillonella* (35 OTUs).

### 3.3 Asthma

**Fig. 3:**
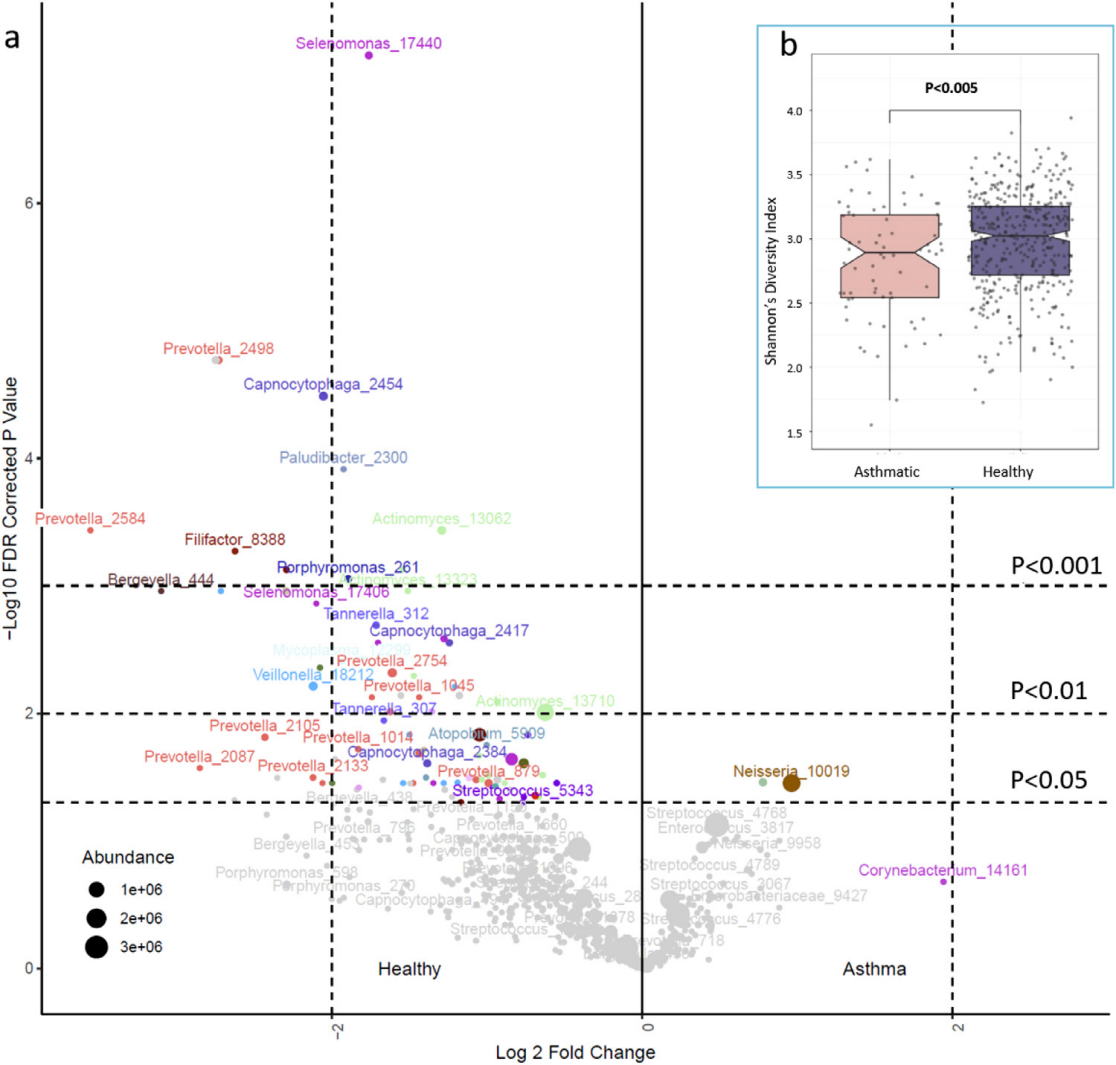
Asthma and the airway microbiome. (a) The volcano plot shows significant differences in the abundance of OTUs between asthmatics and non-smoking subjects with less than 10 packyears of lifetime exposure. Fold change is shown on the x axis and -log10 *P* (FDR corrected) on the y axis. Relative abundances are reflected in the data point sizes; (b) shows differences in alpha diversity between asthmatics and unaffected non-smoking subjects (boxes show inter-quartile range, notches 95% CI of the median, *P* values are two-sided from multiple regression).

Microbial diversity loss in asthmatics compared to non-smoking subjects was qualitatively different to the effects of smoking. DESeq2 analysis showed only two taxa (*Neisseria* and *Rothia* OTUs) to be increased in abundance in asthmatic airways (*P_adjusted_*< 0.05) (Fig. 3, Supplementary Table 6a). Of these, the *Neisseria* OTU was abundant (4.7% of reads in the population) and showed a 2-fold increase, consistent with increases in *Protebacteria* spp. consistently observed in excess by comparisons of asthmatic and normal airways [1,^15^,^16^,49].

Eighty-four OTUs were in relatively low abundance among asthmatic subjects (Fig. 3, Supplementary Table 6b). In marked contrast to smokers, the affected organisms were often in poorly characterized or potentially fastidious genera, including *Leptotrichia, Selenomonas, Megasphaera* and *Capnocytophaga*. Some representatives of the more common genera *Actinomyces, Prevotella* and *Veillonella* were also less abundant.

Inhaled corticosteroids (ICS) are widely used in the maintenance treatment of asthma, and 27 (35%) of our asthmatics were currently using such therapy. Logistic regression analyses showed no independent effect on ICS use from OTUs positively or negatively associated with asthma, or with microbial diversity.

The module eigenvectors did not correlate with the presence of asthma, indicating that the general structure of oropharyngeal microbial communities in asthmatics was preserved (Fig. 2). Nevertheless, the asthma-enriched *Neisseria_10,019* taxon was a hub of the Neisseria guild, which also contained the significantly reduced *Capnocytophagia_2454* (Supplementary Table 4). Other asthma-reduced taxa were concentrated in the Prevotella.1 (containing 57 of the 84 asthma-associated OTUs) and Prevotella.2 (12/84) guilds (Chi^2^ exact test, *P* = 2.8 × 10^−8^). Asthma-associated OTUs were enriched among the most highly connected module members (OR=18.6, *P* = 2.9 × 10^−9^), and so are well positioned to influence host-microbial interactions. The Neisseria, Prevotella.1 and Prevotella.2 guilds thus provide a focus for further understanding of the ecology of asthmatic airway microbiota.

## 4. Discussion

Our study indicates that, in common with other body sites, the healthy airway microbiota are contained within a structured ecosystem. Although bacterial genera and species differ considerably between body surfaces, the main phyla in airway samples (Firmicutes, Bacteriodetes, Actinobacteria, and Proteobacteria) also dominate the human gut [50], skin [51] and vagina [27]. Our tabulation of OTUs and differences in the airway microbiota between smokers, asthmatics, and a control group that has neither, provides an initial basis for the systematic culture and sequencing of the airway microbiota and their eventual management to prevent and treat common respiratory conditions.

Although our results were well powered to map microbial community composition, limited functions could be surmised by genus assignments and the relationship of the networks to each other. Our findings may frame future metagenomic and metatranscriptomic shotgun sequencing, aiding the systematic accrual of reference genomes.

The network analyses captured very strong positive and negative correlations between the abundances of different taxa. They are indicators that the airway microbiota form a complex and highly structured ecosystem. We have named networks according to their most abundant members, but the defining functional traits of the most important networks will await metagenomic analyses. These networks are likely to interact with secreted host factors that either constrain airway pathogens or support commensal bacteria [52]. Such factors have not yet been systematically surveyed.

Cigarette smoking has previously been shown to affect the oral [53] and airway microbiota [4] with characteristic increases in *Streptococcus* spp. The decline in *Neisseria* spp. in our samples (Fig. 1 volcano plot) is also consistent with a loss of Proteobacteria reported in the mouths of smokers [53]. The extent of disruption to the airway ecosystem suggests a significant capacity for the microbiota themselves to damage human health. The loss of diversity and the increase in prevalence of *S. pneumoniae/mitis* clades revealed by *map* gene sequencing may predispose smokers to the recurrent infections that lead to COPD [4,5] and dental disease [53]. Smoking is accompanied by substantial changes in the bowel flora [54] that may mediate smoking influences on inflammatory bowel disease. Bacteria have known roles in the genesis of cancer in general [55] and in lung cancer specifically [6]. *Streptococcus* spp. produce an array of potent toxins that act against human cells or tissues [56], and the expansion of *Streptococcus* clades in smokers might be carcinogenic. Most patients with lung cancer have been heavy smokers and smoking often continues after diagnosis. The gut microbiome influences lung cancer responses to immunotherapy [57], and our results suggest that the local lung microbiota may also modify therapeutic outcomes.

Although the profound consequences of cigarette smoking are clear, the community degradation seen in asthmatics is more subtle and without an obvious cause. Importantly, within asthmatic subjects we did not find current ICS use to be associated with additional microbial abnormalities. The presence or absence of ICS induced changes in the bacterial microbiota is contentious [58], but our results are consistent with controlled studies of steroid and antibiotic naïve wheezing infants [59] and adults with steroid-naive atopic asthma [60]. We were not able to inform on ICS effects in the thoracic airways or in more severe disease.

Divergent (but potentially complementary) theories are offered on mechanisms by which microbial diversity might prevent asthma. The “immune deviation” hypothesis suggests that the adaptive immune system needs exposure to infections in order to avoid inappropriate reactions [61]. An extension of this model is that absence of commensal organisms leads to loss of local or systematic tonic signals that normally down-regulate immune responses at mucosal surfaces [14]. Our findings, of reduced numbers of distinctive low-abundance organisms, are consistent with immune modulation by these organisms.

However, the consistent finding of excesses of *Proteobacteria* in this and other studies [1,^15^,^16^,60] (and *Streptococcus* spp. in severe disease [15,^17^,18]) are also consistent with asthmatic airway inflammation that follows intermittent mucosal damage by bacteria. *Proteobacteria* include many known potential pathogens from the genera *Haemophilus, Moraxella*, and *Neisseria* that, despite the ability to cause disease, are commonly carried without symptoms in the population (“pathobionts”) [2]. In the “asthma as an infection” hypothesis it becomes possible that a diverse microbial community protects against asthma through inhibition of pathobiont effects, by modifying their growth, adherence or biofilm formation [62].

Our study has limitations that may affect interpretation of the results. The results from cross-sectional surveys are descriptive, and detected associations are hypothesis-generating and not necessarily causal. Although the effects of smoking on the microbiota were profound, there was only one diagnosed case of COPD (the late stage of smokers’ lung disease). Asthma was in general mild with a strong atopic component, and the data gathered in this general epidemiological survey do not allow investigation of severe or neutrophilic asthma phenotypes that may exhibit different microbial signatures [14,63].

This single-centre study does not address the level of heterogeneity of airway microbial communities in other environments. The most common taxa appear similar to many published studies of Western subjects in health and disease, but little is known about airway microbiota in the developing world. Direct comparisons will depend on consistency of sample collections and meta-analyses of sequences amplified and analyzed by standardised protocols.

Overall, our results provide a strong impetus to isolate and study the individual organisms that are perturbed in asthmatic airways, and consequently to test in model systems hypotheses that involve immune modulation or mucosal damage.

We suggest that systematic culture, genome sequencing and metabolomic profiling of the airway microbiota are necessary to develop airway metagenomics, and that integrated study of these factors will underpin understanding of the interactions between bacteria, the airway mucosa, and the airway immune system. Such studies may inform whether replacement of specific organisms can offer a strategy for the prevention of asthma.

## Contributors

Overall study design: AWM, AJ, MH, JH, MJC, MFM, and WOCMC. Busselton Survey and sample collection: MH, JH, AJ, AWM. Verification of the underlying data: MFM, MJC, and MH. Microbial analysis strategy (laboratory and bioinformatics); MJC, MFM, PJ, EMT. Laboratory experiments: EMT, with assistance and advice by MJC, PJ, LC and MFM. Primary ecological analyses EMT with input from MJC and PJ. Network analysis EMT and SWO. Secondary analyses WOCC. EMT, MFM and WOCMC wrote the first draft of the paper. All authors have read and contributed to the final version of the paper.

## Declaration of Competing Interest

The authors have no competing interests to declare.

## Appendix Supplementary materials

Image, application 1

Image, application 2

Image, application 3
