# Supplementary material for: Airway microbial communities, smoking and asthma in a general population sample
Source: eBioMedicine. 2021 Aug 20;71:103538. doi: 10.1016/j.ebiom.2021.103538 (PMC8387768; doi:10.1016/j.ebiom.2021.103538)
Supplement: Supplementary file 1 [file mmc1.docx]

Supplementary Information Turek *et al.* 2020

Contents

[Figures 2](#_Toc75804475)

[Supplementary Figure 1. Flow diagram showing stages of data clean-up and analyses for 16S rRNA gene sequences 2](#_Toc75804476)

[Supplementary Figure 2. Flow diagram showing stages of data clean-up and analyses for *map* sequences to distinguish *Streptococcus* spp. 2](#_Toc75804477)

[Supplementary Figure 3. Characteristics of the airway microbiome in a general population 3](#_Toc75804478)

[Supplementary Figure 4. Composition diagram showing subjects for subgroup analyses of smoking and asthma associations. 4](#_Toc75804479)

[Tables 5](#_Toc75804480)

[Supplementary Table 1. Subject characteristics for the 529 individuals with completed 16S analyses 5](#_Toc75804481)

[Supplementary Table 2. Top 100 OTUs in all subjects 6](#_Toc75804482)

[Supplementary Table 3. Stepwise regression for determinants of Shannon alpha diversity (IBM SPSS 25) 10](#_Toc75804483)

[Supplementary Table 4. Weighted Gene Correlation Network Analysis (WGCNA): Principal Module Membership and Hubs 11](#_Toc75804484)

[Supplementary Table 5a. Individual OTUs increased in smokers 20](#_Toc75804485)

[Supplementary Table 5b. OTUs decreased in smokers 20](#_Toc75804486)

[Supplementary Table 6a. OTUs increased in asthmatics 21](#_Toc75804487)

[Supplementary Table 6b. OTUs decreased in asthmatics 21](#_Toc75804488)

[Supplementary Table 7. Analysis of *map* gene: frequencies and identities of *Streptococcus* spp. 22](#_Toc75804489)

[Supplementary Table 8. *Streptococcus* spp. affected by smoking 25](#_Toc75804490)

# Figures

## Supplementary Figure 1. Flow diagram showing stages of data clean-up and analyses for 16S rRNA gene sequences


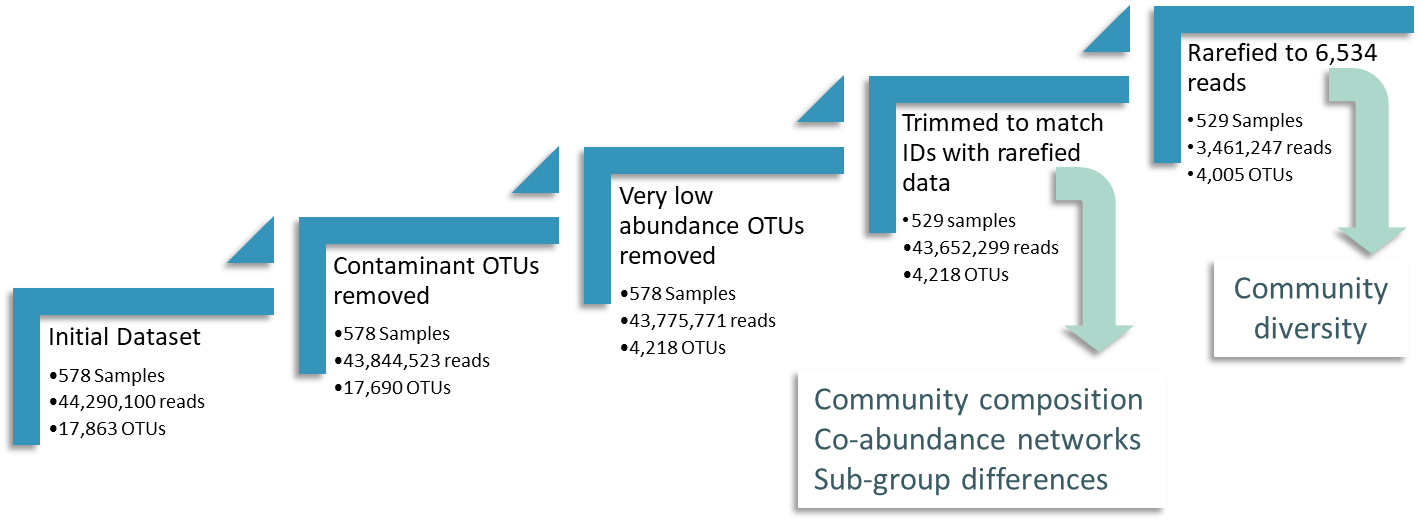


The diagram reads upward from left to right. OTU=operational taxonomic unit, based on 16S RNA gene sequences.

## Supplementary Figure 2. Flow diagram showing stages of data clean-up and analyses for *map* sequences to distinguish *Streptococcus* spp.


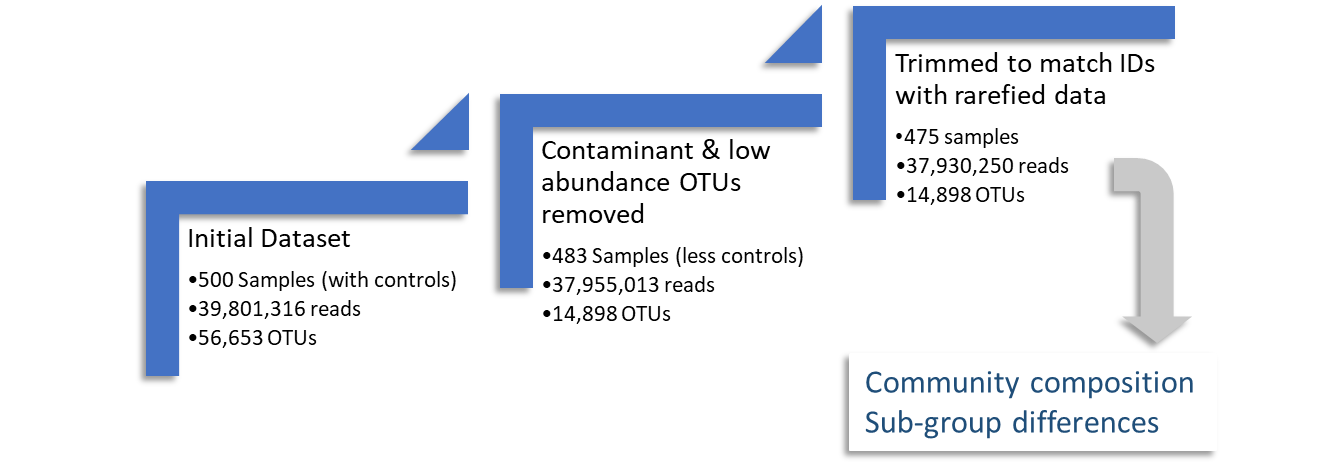


The diagram reads upward from left to right. OTU=operational taxonomic unit, based on *map* gene sequences.

## Supplementary Figure 3. Characteristics of the airway microbiome in a general population


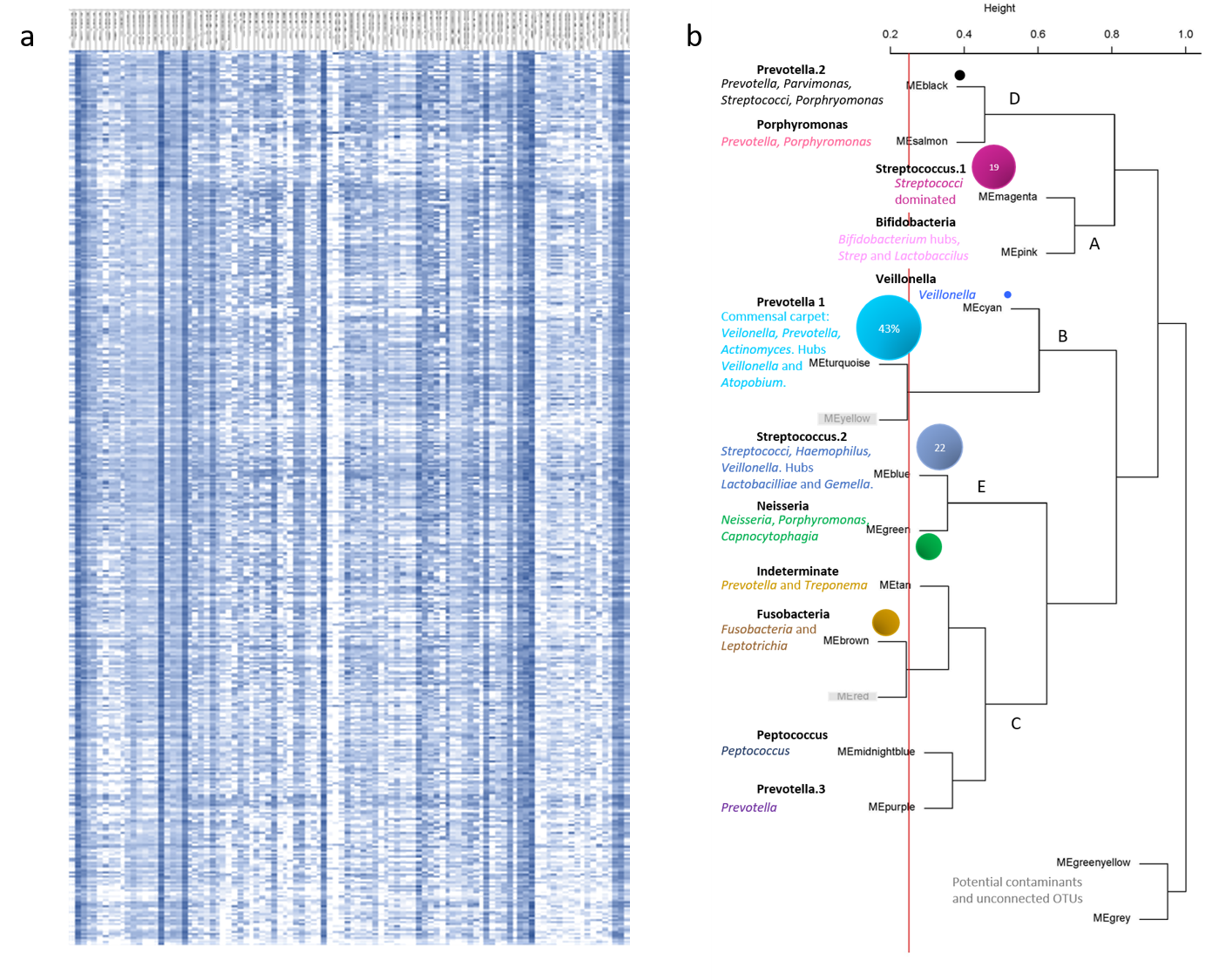


a) Heatmap of the log abundance of the top 100 taxa (OTUs from 16S rRNA sequences) in all subjects. OTUs are shown on the x axis and individual subject results along the y. Community composition is conserved across the population; b) Relationship between WGCNA networks, based on correlation between neighbouring module members. Red and Yellow modules are below the differentiation threshold (red line) and are merged with their immediate neighbours (turquoise and brown respectively). Bacteria not connected to other taxa are in the grey module. Members of the greenyellow module include known contaminants, consistent with the distance from other networks.

## Supplementary Figure 4. Composition diagram showing subjects for subgroup analyses of smoking and asthma associations.


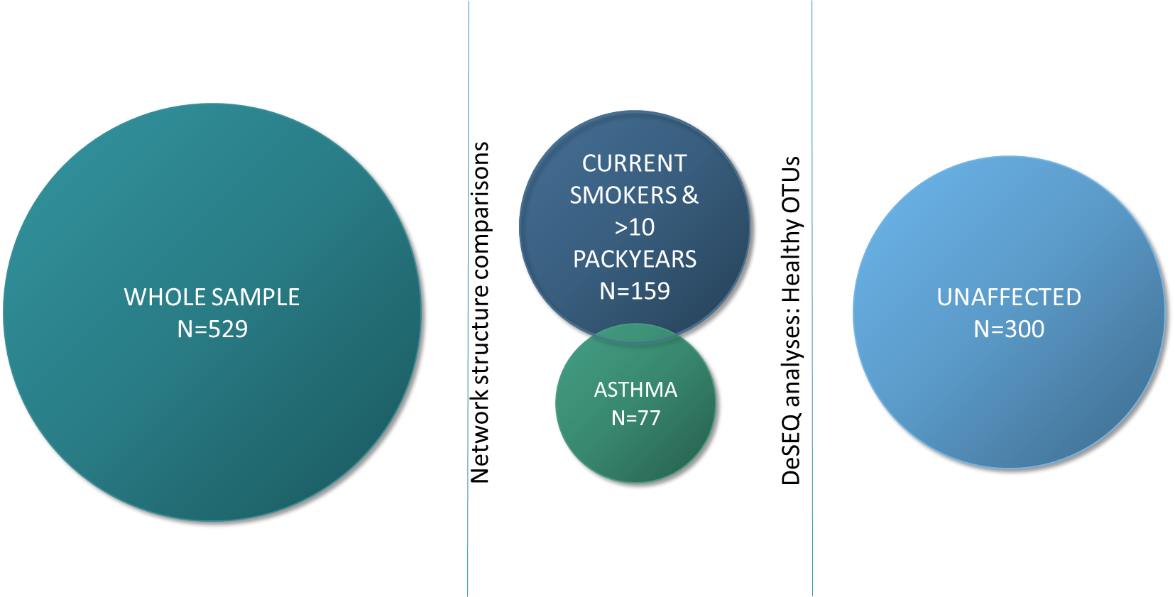


The diagram shows the subjects used in analysing associations with smoking and asthma. WGCNA networks were generated on the whole sample (representing the Busselton population), and then compared with networks from current smokers and from asthmatics. Seven asthmatics were current smokers and were included in network analyses for both groups. To examine OTU associations with disease, abundance differences were tested between smokers or asthmatics against subjects unaffected by these conditions.

# Tables

## Supplementary Table 1. Subject characteristics for the 529 individuals with completed 16S analyses

|  | All (n=529) | Non-asthma non-smokers (n=399) | Asthmatics (n=77)* | Current smokers (n=60) ¶ |
| --- | --- | --- | --- | --- |
| Age (SEM) years | 55.7 (0.24) | 55.9 (0.28) | 54.7 (0.65) | 55.6 (0.72) |
| Sex % Female | 51.0% | 51.8% | 53.0% | 41.7% |
| FEV1% (SEM) | 95.6% (0.6) | 102.5% (0.5) | 95.0% (0.7) | 92.1% (0.7) |
| FVC% (SEM) | 98.7% (0.5) | 101.2% (0.5) | 96.1% (0.6) | 93.0% (0.8) |
| Inhaled steroids | 5.3% | 0.3% | 35.1% | 3.3% |
| Atopy | 49.5% | 44.0% | 75.3% | 55.0% |
| Neutrophils | 3.46 (0.05) | 3.33(0.06) | 3.55 (0.13) | 4.39 (0.17) |
| Eosinophils | 0.194 (0.005) | 0.180 (0.005) | 0.244 (0.02) | 0.225 (0.017) |

* includes 7 current smokers; ¶includes 7 asthmatics.

BMI = Body Mass Index, Atopy = 3mm or greater skin prick test response to mixed grasses or to *D. pteronyssinus* or *D. farinae*, ppFEV baseline = percent predicted forced expiratory volume baseline, ppFVC baseline = percent predicted forced vital capacity baseline

## Supplementary Table 2. Top 100 OTUs in all subjects

| **Row Labels** | **Total Sequences (Abundance)** | **Prevalence (out of 529 subjects)** |
| --- | --- | --- |
| ***Firmicutes*** | **23373367** |  |
| ***Streptococcus*** | **8258368** |  |
| *Streptococcus_20297* | 3019924 | 529 |
| *Streptococcus_20302* | 39744 | 528 |
| *Streptococcus_20338* | 61991 | 527 |
| *Streptococcus_28* | 62335 | 520 |
| *Streptococcus_4768* | 4896262 | 529 |
| *Streptococcus_6617* | 14951 | 517 |
| *Streptococcus_6789* | 23088 | 498 |
| *Streptococcus_6815* | 18701 | 500 |
| *Streptococcus_6916* | 22255 | 525 |
| *Streptococcus_7798* | 64585 | 529 |
| *Streptococcus_7928* | 34532 | 514 |
| ***Veillonella*** | **5999024** |  |
| *Veillonella_16405* | 23284 | 451 |
| *Veillonella_16908* | 19869 | 470 |
| *Veillonella_18212* | 163662 | 380 |
| *Veillonella_19164* | 23913 | 455 |
| *Veillonella_19388* | 97522 | 526 |
| *Veillonella_19389* | 89839 | 527 |
| *Veillonella_19390* | 817686 | 529 |
| *Veillonella_19412* | 4763249 | 529 |
| ***Unidentified_Firmicutes*** | **5146895** |  |
| *Firmicutes_6640* | 5146895 | 529 |
| ***Selenomonas*** | **745942** |  |
| *Selenomonas_17440* | 67889 | 525 |
| *Selenomonas_17559* | 643883 | 529 |
| *Selenomonas_17724* | 34170 | 486 |
| ***Gemella*** | **714904** |  |
| *Gemella_3258* | 714904 | 529 |
| ***Granulicatella*** | **633823** |  |
| *Granulicatella_3873* | 24379 | 441 |
| *Granulicatella_3979* | 609444 | 529 |
| ***Johnsonella*** | **306698** |  |
| *Johnsonella_15517* | 306698 | 495 |
| ***Lachnoanaerobaculum*** | **299622** |  |
| *Lachnoanaerobaculum_14874* | 224079 | 527 |
| *Lachnoanaerobaculum_14972* | 75543 | 516 |
| ***Megasphaera*** | **287130** |  |
| *Megasphaera_16215* | 287130 | 528 |
| ***NA*** | **202588** |  |
| *Clostridiales_8242* | 16636 | 394 |
| *Lactobacillales_3640* | 14800 | 502 |
| *Lactobacillales_3650* | 21695 | 507 |
| *Ruminococcaceae_11741* | 23711 | 411 |
| *Ruminococcaceae_11808* | 35320 | 464 |
| *Veillonellaceae_17891* | 90426 | 410 |
| ***Stomatobaculum*** | **189635** |  |
| *Stomatobaculum_15638* | 81904 | 481 |
| *Stomatobaculum_15766* | 107731 | 501 |
| ***Oribacterium*** | **185734** |  |
| *Oribacterium_15113* | 40336 | 506 |
| *Oribacterium_15121* | 20558 | 391 |
| *Oribacterium_15965* | 124840 | 524 |
| ***Solobacterium*** | **102474** | **525** |
| *Solobacterium_11985* | 102474 | 525 |
| ***Peptostreptococcus*** | **74697** |  |
| *Peptostreptococcus_20146* | 74697 | 486 |
| ***Peptococcus*** | **70217** |  |
| *Peptococcus_9344* | 70217 | 466 |
| ***Parvimonas*** | **68222** |  |
| *Parvimonas_8503* | 68222 | 513 |
| ***Butyrivibrio*** | **45790** |  |
| *Butyrivibrio_14629* | 45790 | 438 |
| ***Catonella*** | **21066** |  |
| *Catonella_9245* | 21066 | 495 |
| ***Filifactor*** | **20538** |  |
| *Filifactor_8388* | 20538 | 299 |
| ***Bacteroidetes*** | **7726297** |  |
| ***Prevotella*** | **6705316** |  |
| *Prevotella_1014* | 18947 | 265 |
| *Prevotella_1525* | 4610220 | 529 |
| *Prevotella_1736* | 28316 | 434 |
| *Prevotella_1750* | 16281 | 397 |
| *Prevotella_1791* | 25413 | 462 |
| *Prevotella_1868* | 351150 | 526 |
| *Prevotella_1969* | 346641 | 519 |
| *Prevotella_2105* | 33863 | 214 |
| *Prevotella_2177* | 22805 | 456 |
| *Prevotella_2498* | 45434 | 380 |
| *Prevotella_2598* | 685534 | 527 |
| *Prevotella_2754* | 168126 | 476 |
| *Prevotella_2890* | 89671 | 446 |
| *Prevotella_621* | 144656 | 495 |
| *Prevotella_822* | 29280 | 376 |
| *Prevotella_879* | 88979 | 494 |
| ***Porphyromonas*** | **631475** |  |
| *Porphyromonas_11896* | 491972 | 519 |
| *Porphyromonas_261* | 54868 | 420 |
| *Porphyromonas_598* | 84635 | 87 |
| ***Capnocytophaga*** | **317872** |  |
| *Capnocytophaga_2384* | 64767 | 399 |
| *Capnocytophaga_2417* | 26121 | 437 |
| *Capnocytophaga_2454* | 113957 | 483 |
| *Capnocytophaga_509* | 113027 | 495 |
| ***Tannerella*** | **38759** |  |
| *Tannerella_312* | 38759 | 430 |
| ***Bergeyella*** | **32875** |  |
| *Bergeyella_430* | 32875 | 490 |
| ***Fusobacteria*** | **3706367** | **2417** |
| ***Fusobacterium*** | **1921431** |  |
| *Fusobacterium_7409* | 1921431 | 529 |
| ***Leptotrichia*** | **1784936** |  |
| *Leptotrichia_8776* | 866757 | 527 |
| *Leptotrichia_8876* | 215430 | 362 |
| *Leptotrichia_8929* | 377771 | 492 |
| *Leptotrichia_8995* | 324978 | 507 |
| ***Proteobacteria*** | **3692787** |  |
| ***Neisseria*** | **2002143** |  |
| *Neisseria_10019* | 1735113 | 525 |
| *Neisseria_10020* | 16590 | 397 |
| *Neisseria_10178* | 15187 | 420 |
| *Neisseria_9888* | 76462 | 428 |
| *Neisseria_9962* | 158791 | 502 |
| ***Haemophilus*** | **1517994** |  |
| *Haemophilus_10797* | 96360 | 516 |
| *Haemophilus_10843* | 35829 | 507 |
| *Haemophilus_11056* | 165096 | 427 |
| *Haemophilus_11091* | 1199062 | 529 |
| *Haemophilus_11369* | 21647 | 392 |
| ***NA*** | **142834** |  |
| *Neisseriaceae_10099* | 19372 | 434 |
| *Pasteurellaceae_11461* | 123462 | 515 |
| ***Campylobacter*** | **29816** |  |
| *Campylobacter_8553* | 29816 | 517 |
| ***Actinobacteria*** | **3130379** |  |
| ***Actinomyces*** | **2014748** |  |
| *Actinomyces_13062* | 152157 | 509 |
| *Actinomyces_13710* | 1862591 | 529 |
| ***Atopobium*** | **921802** |  |
| *Atopobium_5828* | 905892 | 529 |
| *Atopobium_5846* | 15910 | 454 |
| ***Rothia*** | **157077** |  |
| *Rothia_13982* | 87838 | 524 |
| *Rothia_14025* | 69239 | 406 |
| ***Bifidobacterium*** | **36752** |  |
| *Bifidobacterium_13861* | 36752 | 249 |
| ***Candidate_division_TM7*** | **60470** |  |
| ***NA*** | **60470** |  |
| *Candidate_division_TM7_12053* | 60470 | 477 |
| ***Candidate_division_SR1*** | **49511** |  |
| ***(blank)*** | **49511** |  |
| *Candidate_division_SR1_12158* | 49511 | 341 |
| **Grand Total** | **41739178** |  |

## Supplementary Table 3. Stepwise regression for determinants of Shannon alpha diversity (IBM SPSS 25)

| **Model Summary** | | | | | |
| --- | --- | --- | --- | --- | --- |
| Model | Change Statistics | | | | |
|  | R Square Change | F Change | df1 | df2 | Sig. F Change |
| 1 | .060^a^ | 33.005 | 1 | 515 | .000 |
| 2 | .014^b^ | 7.905 | 1 | 514 | .005 |
| 3 | .008^c^ | 4.323 | 1 | 513 | .038 |
| a. Predictors: (Constant), Current Smoking | | | | | |
| b. Predictors: (Constant), Current Smoking, Asthma | | | | | |
| c. Predictors: (Constant), Current Smoking, Asthma, Packyears | | | | | |

| **Coefficients^a^** | | | | | | | | |
| --- | --- | --- | --- | --- | --- | --- | --- | --- |
| Model | | Unstandardized Coefficients | | Standardized Coefficients | t | Sig. | 95% Confidence Interval for B | |
|  |  | B | Std. Error | Beta |  |  | Lower Bound | Upper Bound |
| 1 | (Constant) | 2.710 | .017 |  | 155.278 | .000 | 2.675 | 2.744 |
|  | Current Smoking | -.294 | .051 | -.245 | -5.745 | .000 | -.395 | -.194 |
| 2 | (Constant) | 2.729 | .019 |  | 146.186 | .000 | 2.693 | 2.766 |
|  | Current Smoking | -.301 | .051 | -.251 | -5.903 | .000 | -.401 | -.201 |
|  | Asthma | -.131 | .047 | -.119 | -2.812 | .005 | -.223 | -.039 |
| 3 | (Constant) | 2.744 | .020 |  | 137.569 | .000 | 2.705 | 2.783 |
|  | Current Smoking | -.239 | .059 | -.199 | -4.071 | .000 | -.355 | -.124 |
|  | Asthma | -.130 | .046 | -.119 | -2.809 | .005 | -.222 | -.039 |
|  | Packyears | -.002 | .001 | -.102 | -2.079 | .038 | -.005 | .000 |
| a. Dependent Variable: Shannon alpha diversity index | | | | | | | | |

## Supplementary Table 4. Weighted Gene Correlation Network Analysis (WGCNA): Principal Module Membership and Hubs

Colour shading for Abundance % (the % of reads for the OTU) is derived from the whole dataset and varies from green (highest) to red (lowest). Prevalence % is shown in blue (high) to red (low). Network hubs are highlighted in pink.

| **TurquoiseModule: Prevotella.1** | |  |  |  |  |  |
| --- | --- | --- | --- | --- | --- | --- |
| **OTU_Name** | **Genus** | **Abundance%** | **Prevalence%** | **-log10*(P)* MM** | **-log10*(P)* Smo** | **-log10*(P)* Asth** |
| Veillonella_19412 | Veillonella | 10.91 | 100.00 | 65.05 |  |  |
| Prevotella_1525 | Prevotella | 10.56 | 100.00 | 116.33 |  |  |
| Actinomyces_13710 | Actinomyces | 4.27 | 100.00 | 98.11 |  | 2.01 |
| Atopobium_5828 | Atopobium | 2.08 | 100.00 | 111.68 |  |  |
| Leptotrichia_8776 | Leptotrichia | 1.99 | 99.62 | 83.12 |  | 1.83 |
| Prevotella_2598 | Prevotella | 1.57 | 99.62 | 47.46 |  |  |
| Selenomonas_17559 | Selenomonas | 1.48 | 100.00 | 105.02 |  | 1.64 |
| Leptotrichia_8929 | Leptotrichia | 0.87 | 93.01 | 48.22 |  |  |
| Prevotella_1868 | Prevotella | 0.80 | 99.43 | 130.59 |  |  |
| Prevotella_1969 | Prevotella | 0.79 | 98.11 | 73.41 |  |  |
| Megasphaera_16215 | Megasphaera | 0.66 | 99.81 | 115.22 |  | 1.61 |
| Lachnoanaerobaculum_14874 | Lachnoanaerobaculum | 0.51 | 99.62 | 107.51 |  |  |
| Veillonella_19417 | Veillonella | 0.02 | 79.96 | 173.84 |  |  |
| Atopobium_5926 | Atopobium | 0.03 | 76.37 | 170.20 |  |  |
| Atopobium_5846 | Atopobium | 0.04 | 85.82 | 165.26 |  |  |
| Actinomyces_13214 | Actinomyces | 0.01 | 81.29 | 160.11 |  |  |
| Veillonella_19164 | Veillonella | 0.05 | 86.01 | 158.08 |  |  |
| Veillonella_16405 | Veillonella | 0.05 | 85.26 | 157.79 |  |  |
| Veillonella_16908 | Veillonella | 0.05 | 88.85 | 157.02 |  |  |
| Veillonella_18660 | Veillonella | 0.01 | 78.45 | 151.70 |  |  |
| Veillonella_16572 | Veillonella | 0.03 | 85.26 | 150.10 |  |  |
| Veillonella_17001 | Veillonella | 0.02 | 73.72 | 144.91 |  |  |
|  |  |  |  |  |  |  |
|  |  |  |  |  |  |  |
|  |  |  |  |  |  |  |
| **BlueModule: Streptococcus.2** |  |  |  |  |  |  |
| **OTU_Name** | **Genus** | **Abundance%** | **Prevalence%** | **-log10*(P)* MM** | **-log10*(P)* Smo** | **-log10*(P)* Asth** |
| Streptococcus_4768 | Streptococcus | 11.22 | 100.00 | 61.10 |  |  |
| Haemophilus_11091 | Haemophilus | 2.75 | 100.00 | 90.16 |  |  |
| Veillonella_19390 | Veillonella | 1.87 | 100.00 | 48.69 |  |  |
| Gemella_3258 | Gemella | 1.64 | 100.00 | 97.37 |  |  |
| Granulicatella_3979 | Granulicatella | 1.40 | 100.00 | 109.38 |  |  |
| Haemophilus_11056 | Haemophilus | 0.38 | 80.72 | 14.71 |  |  |
| Pasteurellaceae_11461 | Unknown | 0.28 | 97.35 | 46.26 |  |  |
| Veillonella_19388 | Veillonella | 0.22 | 99.43 | 48.13 |  |  |
| Haemophilus_10797 | Haemophilus | 0.22 | 97.54 | 34.24 |  |  |
| Veillonella_19389 | Veillonella | 0.21 | 99.62 | 57.86 |  |  |
| Streptococcus_4755 | Streptococcus | 0.03 | 99.62 | 66.34 |  |  |
| Rothia_13982 | Rothia | 0.20 | 99.05 | 50.22 |  |  |
| Streptococcus_4687 | Streptococcus | 0.03 | 98.87 | 63.44 |  |  |
| Lactobacillales_4201 | Unknown | 0.03 | 92.82 | 168.95 |  |  |
| Streptococcus_7928 | Streptococcus | 0.08 | 97.16 | 156.20 |  |  |
| Gemella_3389 | Gemella | 0.01 | 87.71 | 150.50 |  |  |
| Gemella_3253 | Gemella | 0.03 | 94.14 | 149.91 |  |  |
| Bacillales_3452 | Unknown | 0.02 | 87.15 | 149.80 |  |  |
| Bacillales_3375 | Unknown | 0.01 | 75.43 | 149.55 |  |  |
| Granulicatella_3474 | Granulicatella | 0.01 | 74.10 | 142.50 |  |  |
| Streptococcus_6167 | Streptococcus | 0.02 | 91.68 | 137.98 |  |  |
| Gemella_3415 | Gemella | 0.02 | 87.71 | 135.65 |  |  |
| Gemella_3297 | Gemella | 0.01 | 77.13 | 131.99 |  |  |
|  |  |  |  |  |  |  |
|  |  |  |  |  |  |  |
|  |  |  |  |  |  |  |
|  |  |  |  |  |  |  |
|  |  |  |  |  |  |  |
|  |  |  |  |  |  |  |
| **MagentaModule: Streptococcus.1** | |  |  |  |  |  |
| **OTU_Name** | **Genus** | **Abundance%** | **Prevalence%** | **-log10*(P)* MM** | **-log10*(P)* Smo** | **-log10*(P)* Asth** |
| Firmicutes_6640 | Unidentified_Firmicutes | 11.79 | 100.00 | 98.27 |  |  |
| Streptococcus_20297 | Streptococcus | 6.92 | 100.00 | 241.97 | 5.52 |  |
| Streptococcus_7798 | Streptococcus | 0.15 | 100.00 | 42.85 |  |  |
| Streptococcus_20338 | Streptococcus | 0.14 | 99.62 | 140.61 |  |  |
| Streptococcus_20302 | Streptococcus | 0.09 | 99.81 | 265.10 | 4.47 |  |
| Streptococcus_6916 | Streptococcus | 0.05 | 99.24 | 146.74 |  |  |
| Streptococcus_6815 | Streptococcus | 0.04 | 94.52 | 88.55 |  |  |
| Streptococcus_5304 | Streptococcus | 0.03 | 99.62 | 148.90 |  |  |
| Streptococcus_20310 | Streptococcus | 0.02 | 96.98 | 205.36 |  |  |
| Rothia_13888 | Rothia | 0.02 | 90.93 | 26.77 |  |  |
| Streptococcus_8021 | Streptococcus | 0.01 | 96.41 | 164.18 |  |  |
| Streptococcus_20305 | Streptococcus | 0.02 | 95.27 | 152.52 |  |  |
| Streptococcus_20337 | Streptococcus | 0.01 | 95.09 | 155.30 |  |  |
| Streptococcus_7171 | Streptococcus | 0.01 | 94.14 | 130.37 |  |  |
|  |  |  |  |  |  |  |
| **BrownModule: Fusobacteria** |  |  |  |  |  |  |
| **OTU_Name** | **Genus** | **Abundance%** | **Prevalence%** | **-log10*(P)* MM** | **-log10*(P)* Smo** | **-log10*(P)* Asth** |
| Fusobacterium_7409 | Fusobacterium | 4.40 | 100.00 | 149.82 | 6.27 |  |
| Leptotrichia_8995 | Leptotrichia | 0.74 | 95.84 | 120.71 | 7.16 |  |
| Prevotella_621 | Prevotella | 0.33 | 93.57 | 78.30 |  |  |
| Oribacterium_15965 | Oribacterium | 0.29 | 99.05 | 69.50 |  |  |
| Stomatobaculum_15638 | Stomatobaculum | 0.19 | 90.93 | 77.39 |  |  |
| Lachnoanaerobaculum_14972 | Lachnoanaerobaculum | 0.17 | 97.54 | 99.28 |  |  |
| Peptostreptococcus_20146 | Peptostreptococcus | 0.17 | 91.87 | 95.44 |  |  |
| Prevotella_2498 | Prevotella | 0.10 | 71.83 | 21.89 |  |  |
| Tannerella_312 | Tannerella | 0.09 | 81.29 | 74.55 |  |  |
| Prevotella_1736 | Prevotella | 0.06 | 82.04 | 94.58 |  |  |
| Catonella_9245 | Catonella | 0.05 | 93.57 | 82.14 |  |  |
| Clostridiales_8204 | Unknown | 0.02 | 83.18 | 51.25 |  |  |
| Capnocytophaga_2417 | Capnocytophaga | 0.06 | 82.61 | 35.61 |  | 2.55 |
| Leptotrichia_8999 | Leptotrichia | 0.03 | 64.46 | 115.42 |  |  |
| Prevotella_660 | Prevotella | 0.01 | 55.58 | 93.98 |  |  |
| Peptostreptococcus_20150 | Peptostreptococcus | 0.01 | 55.58 | 89.53 |  |  |
| Peptostreptococcus_20149 | Peptostreptococcus | 0.01 | 55.58 | 84.71 |  |  |
| Leptotrichia_8997 | Leptotrichia | 0.00 | 39.51 | 83.14 |  |  |
|  |  |  |  |  |  |  |
| **GreenModule: Neisseria** |  |  |  |  |  |  |
| **OTU_Name** | **Genus** | **Abundance%** | **Prevalence%** | **-log10*(P)* MM** | **-log10*(P)* Smo** | **-log10*(P)* Asth** |
| Neisseria_10019 | Neisseria | 3.97 | 99.24 | 243.35 | 15.16 |  |
| Porphyromonas_11896 | Porphyromonas | 1.13 | 98.11 | 83.71 |  |  |
| Neisseria_9962 | Neisseria | 0.36 | 94.90 | 119.40 |  |  |
| Capnocytophaga_2454 | Capnocytophaga | 0.26 | 91.30 | 43.16 |  | 4.49 |
| Capnocytophaga_509 | Capnocytophaga | 0.26 | 93.57 | 62.39 |  |  |
| Neisseria_9888 | Neisseria | 0.18 | 80.91 | 113.68 |  |  |
| Capnocytophaga_2384 | Capnocytophaga | 0.15 | 75.43 | 36.66 |  | 1.61 |
| Bergeyella_430 | Bergeyella | 0.08 | 92.63 | 83.05 |  |  |
| Haemophilus_11369 | Haemophilus | 0.05 | 74.10 | 206.44 |  |  |
| Neisseriaceae_10099 | Unknown | 0.04 | 82.04 | 201.65 |  |  |
| Neisseria_10209 | Neisseria | 0.03 | 85.44 | 74.46 |  |  |
| Neisseria_10178 | Neisseria | 0.03 | 79.40 | 219.70 |  |  |
| Neisseria_10020 | Neisseria | 0.04 | 75.05 | 230.93 | 12.58 |  |
| Neisseria_14366 | Neisseria | 0.03 | 71.27 | 209.30 |  |  |
| Neisseria_10062 | Neisseria | 0.02 | 69.00 | 202.89 |  |  |
| Neisseria_9813 | Neisseria | 0.01 | 57.47 | 153.62 |  |  |
| Neisseria_10095 | Neisseria | 0.01 | 56.33 | 153.60 |  |  |
| Neisseria_9715 | Neisseria | 0.00 | 52.17 | 128.68 |  |  |
|  |  |  |  |  |  |  |
|  |  |  |  |  |  |  |
|  |  |  |  |  |  |  |
|  |  |  |  |  |  |  |
| **BlackModule: Prevotella.2** |  |  |  |  |  |  |
| **OTU_Name** | **Genus** | **Abundance%** | **Prevalence%** | **-log10*(P)* MM** | **-log10*(P)* Smo** | **-log10*(P)* Asth** |
| Parvimonas_8503 | Parvimonas | 0.16 | 96.98 | 78.19 |  |  |
| Streptococcus_28 | Streptococcus | 0.14 | 98.30 | 83.83 |  |  |
| Porphyromonas_261 | Porphyromonas | 0.13 | 79.40 | 84.33 |  |  |
| Prevotella_1791 | Prevotella | 0.06 | 87.33 | 92.42 |  |  |
| Prevotella_2177 | Prevotella | 0.05 | 86.20 | 79.98 |  |  |
| Filifactor_8388 | Filifactor | 0.05 | 56.52 | 75.32 |  |  |
| Prevotella_1014 | Prevotella | 0.04 | 50.09 | 46.45 |  |  |
| Clostridiales_8242 | Unknown | 0.04 | 74.48 | 110.42 | 3.85 |  |
| Fusobacterium_7664 | Fusobacterium | 0.03 | 12.10 | 11.89 |  |  |
| Veillonellaceae_19943 | Unknown | 0.02 | 65.60 | 95.50 |  |  |
| Paludibacter_2300 | Paludibacter | 0.01 | 71.27 | 31.48 |  |  |
| Mycoplasma_12299 | Mycoplasma | 0.01 | 58.60 | 58.47 |  |  |
| Tannerella_307 | Tannerella | 0.01 | 52.17 | 72.49 |  |  |
| Synergistaceae_8518 | Unknown | 0.00 | 38.00 | 68.89 |  |  |
|  |  |  |  |  |  |  |
| **CyanModule: Veillonella** |  |  |  |  |  |  |
| **OTU_Name** | **Genus** | **Abundance%** | **Prevalence%** | **-log10*(P)* MM** | **-log10*(P)* Smo** | **-log10*(P)* Asth** |
| Veillonella_18212 | Veillonella | 0.37 | 71.83 | 131.39 | 6.76 |  |
| Veillonella_19620 | Veillonella | 0.00 | 34.78 | 97.93 | 2.22 |  |
| Firmicutes_18412 | Unknown | 0.00 | 23.06 | 80.79 |  |  |
| Firmicutes_18411 | Unknown | 0.00 | 22.68 | 78.64 |  |  |
| Veillonella_18239 | Veillonella | 0.00 | 14.74 | 47.61 |  |  |
| Firmicutes_18226 | Unknown | 0.00 | 16.45 | 57.42 |  |  |
| Veillonella_18283 | Veillonella | 0.00 | 12.10 | 40.64 |  |  |
| Clostridiales_18253 | Unknown | 0.00 | 15.50 | 50.33 |  |  |
| Atopobium_5807 | Atopobium | 0.00 | 16.07 | 45.32 |  |  |
| Veillonella_18222 | Veillonella | 0.00 | 12.48 | 41.61 |  |  |
| Veillonella_19619 | Veillonella | 0.00 | 19.66 | 31.64 |  |  |
| Veillonella_19463 | Veillonella | 0.00 | 18.15 | 41.44 |  |  |
| Veillonella_19991 | Veillonella | 0.00 | 17.58 | 40.93 |  |  |
| Veillonella_18289 | Veillonella | 0.00 | 12.67 | 41.53 |  |  |
|  |  |  |  |  |  |  |
| **PurpleModule: Prevotella.3** |  |  |  |  |  |  |
| **OTU_Name** | **Genus** | **Abundance%** | **Prevalence%** | **-log10*(P)* MM** | **-log10*(P)* Smo** | **-log10*(P)* Asth** |
| Prevotella_2890 | Prevotella | 0.21 | 84.31 | 117.63 |  |  |
| RF9_12250 |  | 0.01 | 18.71 | 22.85 |  |  |
| Prevotella_1442 | Prevotella | 0.00 | 26.09 | 67.50 |  |  |
| Prevotella_2912 | Prevotella | 0.00 | 25.52 | 65.56 |  |  |
| Clostridiales_8287 | Unknown | 0.00 | 31.57 | 33.68 |  |  |
| Capnocytophaga_499 | Capnocytophaga | 0.00 | 15.88 | 15.09 |  |  |
| Prevotella_2816 | Prevotella | 0.00 | 17.96 | 44.24 |  |  |
| Prevotella_2891 | Prevotella | 0.00 | 18.53 | 49.62 |  |  |
| Prevotella_1431 | Prevotella | 0.00 | 18.90 | 47.71 |  |  |
| Prevotella_2146 | Prevotella | 0.00 | 16.64 | 45.84 |  |  |
| Prevotella_773 | Prevotella | 0.00 | 21.93 | 30.55 |  |  |
| Prevotella_2732 | Prevotella | 0.00 | 16.45 | 41.06 |  |  |
| Prevotella_2913 | Prevotella | 0.00 | 10.59 | 32.28 |  |  |
|  |  |  |  |  |  |  |
| **TanModule: Indeterminate** |  |  |  |  |  |  |
| **OTU_Name** | **Genus** | **Abundance%** | **Prevalence%** | **-log10*(P)* MM** | **-log10*(P)* Smo** | **-log10*(P)* Asth** |
| Candidatedivision_SR1_12158 |  | 0.11 | 64.46 | 73.85 |  |  |
| Prevotella_822 | Prevotella | 0.07 | 71.08 | 123.87 | 3.27 |  |
| RF9_12187 |  | 0.01 | 34.59 | 35.69 |  |  |
| Candidatedivision_SR1_12141 |  | 0.01 | 24.39 | 41.68 |  |  |
| Treponema_11646 | Treponema | 0.01 | 33.46 | 46.11 |  |  |
| Prevotella_1354 | Prevotella | 0.00 | 29.11 | 75.12 | 1.89 |  |
| Prevotella_809 | Prevotella | 0.00 | 23.82 | 66.19 |  |  |
| Treponema_11722 | Treponema | 0.00 | 14.56 | 29.96 |  |  |
| Prevotella_848 | Prevotella | 0.00 | 16.64 | 48.81 |  |  |
| Candidatedivision_SR1_12142 |  | 0.00 | 9.83 | 20.74 |  |  |
| Prevotella_2653 | Prevotella | 0.00 | 17.58 | 52.94 |  |  |
| Prevotella_640 | Prevotella | 0.00 | 15.12 | 43.37 |  |  |
| Prevotella_858 | Prevotella | 0.00 | 15.12 | 44.50 |  |  |
|  |  |  |  |  |  |  |
| **SalmonModule: Porphymonas** | |  |  |  |  |  |
| **OTU_Name** | **Genus** | **Abundance%** | **Prevalence%** | **-log10*(P)* MM** | **-log10*(P)* Smo** | **-log10*(P)* Asth** |
| Porphyromonas_598 | Porphyromonas | 0.19 | 16.45 | 22.20 |  |  |
| Prevotella_1923 | Prevotella | 0.00 | 15.31 | 34.53 | 2.52 |  |
| Bacteroides_612 | Bacteroides | 0.00 | 0.38 | 1.88 |  |  |
| Prevotella_992 | Prevotella | 0.00 | 10.78 | 33.09 |  |  |
| Prevotella_1003 | Prevotella | 0.00 | 0.76 | 2.40 |  |  |
| Lachnospiraceae_15543 | Unknown | 0.00 | 3.97 | 8.30 |  |  |
| Porphyromonas_262 | Porphyromonas | 0.00 | 11.34 | 28.07 |  |  |
| Porphyromonas_588 | Porphyromonas | 0.00 | 1.32 | 4.83 |  |  |
| Desulfovibrio_12642 | Desulfovibrio | 0.00 | 1.89 | 5.07 |  |  |
| Akkermansia_12380 | Akkermansia | 0.00 | 0.38 | 1.95 |  |  |
| Porphyromonas_2279 | Porphyromonas | 0.00 | 7.75 | 16.86 |  |  |
| Porphyromonas_11948 | Porphyromonas | 0.00 | 7.37 | 16.33 |  |  |
| Porphyromonas_2284 | Porphyromonas | 0.00 | 6.24 | 18.04 |  |  |
| Prevotella_1016 | Prevotella | 0.00 | 5.67 | 14.79 |  |  |
| Prevotella_1008 | Prevotella | 0.00 | 5.48 | 16.29 |  |  |
| Prevotella_1319 | Prevotella | 0.00 | 5.48 | 15.80 |  |  |
|  |  |  |  |  |  |  |
| **PinkModule: Bifidobacteria** |  |  |  |  |  |  |
| **OTU_Name** | **Genus** | **Abundance%** | **Prevalence%** | **-log10*(P)* MM** | **-log10*(P)* Smo** | **-log10*(P)* Asth** |
| Bifidobacterium_13861 | Bifidobacterium | 0.08 | 47.07 | 42.25 | 14.06 |  |
| Streptococcus_4578 | Streptococcus | 0.02 | 60.49 | 36.18 |  |  |
| Lactobacillus_4348 | Lactobacillus | 0.01 | 11.34 | 24.34 |  |  |
| Streptococcus_226 | Streptococcus | 0.01 | 16.82 | 14.61 |  |  |
| Lactobacillus_4496 | Lactobacillus | 0.01 | 17.77 | 29.17 |  |  |
| Lactobacillus_4421 | Lactobacillus | 0.01 | 18.90 | 32.18 |  |  |
| Bifidobacteriaceae_13837 | Unknown | 0.01 | 35.92 | 33.08 |  |  |
| Prevotella_1947 | Prevotella | 0.01 | 7.37 | 5.79 |  |  |
| Lactobacillus_4457 | Lactobacillus | 0.00 | 6.24 | 14.67 |  |  |
| Streptococcus_4601 | Streptococcus | 0.00 | 29.11 | 33.12 |  |  |
| Bifidobacteriaceae_13749 | Unknown | 0.00 | 18.90 | 29.72 |  |  |
| Veillonella_20045 | Veillonella | 0.00 | 18.71 | 15.40 |  |  |
| Bifidobacterium_13401 | Bifidobacterium | 0.00 | 16.45 | 37.23 | 10.40 |  |
| Bifidobacterium_13785 | Bifidobacterium | 0.00 | 14.56 | 31.50 |  |  |
|  |  |  |  |  |  |  |
| **MidnightBlueModule: Peptococcus** | |  |  |  |  |  |
| **OTU_Name** | **Genus** | **Abundance%** | **Prevalence%** | **-log10*(P)* MM** | **-log10*(P)* Smo** | **-log10*(P)* Asth** |
| Peptococcus_9344 | Peptococcus | 0.16 | 88.09 | 202.84 | 4.14 |  |
| Peptococcus_9267 | Peptococcus | 0.01 | 47.07 | 133.05 | 4.35 |  |
| Firmicutes_16800 | Unknown | 0.00 | 32.51 | 90.04 |  |  |
| Peptococcus_9274 | Peptococcus | 0.00 | 29.11 | 88.23 |  |  |
| Peptococcus_9298 | Peptococcus | 0.00 | 27.79 | 81.17 |  |  |
| Peptococcus_9351 | Peptococcus | 0.00 | 17.01 | 48.58 |  |  |
| Veillonellaceae_18178 | Unknown | 0.00 | 24.39 | 55.69 |  |  |
| Peptococcus_9291 | Peptococcus | 0.00 | 18.53 | 45.76 |  |  |
| Veillonella_18183 | Veillonella | 0.00 | 21.36 | 59.61 |  |  |
| Peptococcus_9371 | Peptococcus | 0.00 | 17.77 | 45.34 |  |  |
| Peptococcus_9299 | Peptococcus | 0.00 | 20.60 | 52.84 |  |  |
| Streptococcus_5498 | Streptococcus | 0.00 | 20.23 | 51.36 |  |  |
| Veillonella_18513 | Veillonella | 0.00 | 18.71 | 46.91 |  |  |
| Peptococcus_9337 | Peptococcus | 0.00 | 17.77 | 50.04 |  |  |
|  |  |  |  |  |  |  |
| **GreenYellowModule: Contaminants** | |  |  |  |  |  |
| **OTU_Name** | **Genus** | **Abundance%** | **Prevalence%** | **-log10*(P)* MM** | **-log10*(P)* Smo** | **-log10*(P)* Asth** |
| Haemophilus_11389 | Haemophilus | 0.01 | 50.47 | 109.82 | 2.75 |  |
| Neisseria_9743 | Neisseria | 0.00 | 32.14 | 76.18 | 3.21 |  |
| Herbaspirillum_10515 | Herbaspirillum | 0.00 | 23.06 | 71.10 |  |  |
| Herbaspirillum_10738 | Herbaspirillum | 0.00 | 13.99 | 47.11 |  |  |
| Haemophilus_11362 | Haemophilus | 0.00 | 14.56 | 29.66 |  |  |
| Streptococcus_5394 | Streptococcus | 0.00 | 15.69 | 43.41 |  |  |
| Neisseria_9863 | Neisseria | 0.00 | 8.88 | 26.43 |  |  |
| Haemophilus_11176 | Haemophilus | 0.00 | 13.42 | 36.60 |  |  |
| Pseudomonas_12457 | Pseudomonas | 0.00 | 10.59 | 32.97 |  |  |
| Veillonellaceae_16762 | Unknown | 0.00 | 11.53 | 34.35 |  |  |
| Actinomyces_13512 | Actinomyces | 0.00 | 15.31 | 19.99 |  |  |
| Firmicutes_5407 | Unknown | 0.00 | 11.72 | 36.36 |  |  |
| Pseudomonas_12488 | Pseudomonas | 0.00 | 10.78 | 35.41 |  |  |
|  |  |  |  |  |  |  |
| **GreyModule: Unconnected** |  |  |  |  |  |  |
| **OTU_Name** | **Genus** | **Abundance%** | **Prevalence%** |  |  |  |
| Vibrio_11550 | Vibrio | 0.00 | 2.27 |  |  |  |
| Lactobacillales_3493 | Unknown | 0.00 | 0.76 |  |  |  |
| Corynebacterium_14237 | Corynebacterium | 0.00 | 2.08 |  |  |  |
| Comamonadaceae_10394 | Unknown | 0.00 | 1.89 |  |  |  |
| Escherichia_Shigella_9556 | Escherichia_Shigella | 0.00 | 1.32 |  |  |  |
| Corynebacterium_14199 | Corynebacterium | 0.00 | 2.65 |  |  |  |
| Pseudomonadaceae_12534 | Unknown | 0.00 | 1.89 |  |  |  |
| Burkholderia_10482 | Burkholderia | 0.00 | 1.70 |  |  |  |
| Enterobacter_9544 | Enterobacter | 0.00 | 1.70 |  |  |  |
| vadinBB60_12276 | Unknown | 0.00 | 0.38 |  |  |  |
| Haemophilus_10769 | Haemophilus | 0.00 | 2.08 |  |  |  |
| Vibrio_11520 | Vibrio | 0.00 | 1.89 |  |  |  |
| Pasteurellaceae_11449 | Unknown | 0.00 | 1.51 |  |  |  |

## Supplementary Table 5a. Individual OTUs increased in smokers

| **OUT_ID** | **Genus** | **Fold_change** | **-log10(*P*)** | **Abundance** | **Abundance %** | **Prevalence %** | **Change** | **Increase** |
| --- | --- | --- | --- | --- | --- | --- | --- | --- |
| Firmicutes_6640 | Unidentified_Firmicutes | 1.20 | 10.74 | 5146895 | 11.79% | 100.00 | 11846325 | 6,699,430 |
| Veillonella_19412 | Veillonella | 0.70 | 4.60 | 4763249 | 10.91% | 100.00 | 7723612 | 2,960,363 |
| Streptococcus_20297 | Streptococcus | 1.46 | 27.96 | 3019924 | 6.92% | 100.00 | 8285093 | 5,265,169 |
| Rothia_14025 | Rothia | 1.93 | 4.87 | 69239 | 0.16% | 76.75 | 263551 | 194,312 |
| Streptococcus_28 | Streptococcus | 1.19 | 3.71 | 62335 | 0.14% | 98.30 | 142258 | 79,923 |
| Streptococcus_20338 | Streptococcus | 1.13 | 12.02 | 61991 | 0.14% | 99.62 | 135873 | 73,882 |
| Streptococcus_20302 | Streptococcus | 1.40 | 25.63 | 39744 | 0.09% | 99.81 | 104758 | 65,014 |
| Bifidobacterium_13861 | Bifidobacterium | 3.17 | 5.14 | 36752 | 0.08% | 47.07 | 330810 | 294,058 |
| Prevotella_1791 | Prevotella | 1.35 | 3.72 | 25413 | 0.06% | 87.33 | 65005 | 39,592 |
| Streptococcus_6916 | Streptococcus | 1.05 | 6.00 | 22255 | 0.05% | 99.24 | 46124 | 23,869 |
| Streptococcus_6617 | Streptococcus | 0.69 | 4.48 | 14951 | 0.03% | 97.73 | 24172 | 9,221 |
| Streptococcus_5304 | Streptococcus | 1.14 | 14.57 | 14557 | 0.03% | 99.62 | 32024 | 17,467 |
| Actinomyces_13500 | Actinomyces | 0.92 | 4.63 | 13457 | 0.03% | 96.41 | 25494 | 12,037 |
| Streptococcus_4578 | Streptococcus | 2.66 | 7.38 | 10289 | 0.02% | 60.49 | 64849 | 54,560 |
| Bifidobacteriaceae_13811 | Unknown | 2.93 | 5.30 | 10032 | 0.02% | 42.53 | 76595 | 66,563 |
| Streptococcus_20310 | Streptococcus | 1.16 | 12.62 | 9713 | 0.02% | 96.98 | 21691 | 11,978 |
| Streptococcus_4835 | Streptococcus | 0.65 | 3.74 | 9083 | 0.02% | 95.46 | 14228 | 5,145 |
| Rothia_13888 | Rothia | 1.98 | 14.51 | 8711 | 0.02% | 90.93 | 34480 | 25,769 |
| Streptococcus_20305 | Streptococcus | 1.26 | 12.49 | 6569 | 0.02% | 95.27 | 15743 | 9,174 |

## Supplementary Table 5b. OTUs decreased in smokers

| **OUT_ID** | **Genus** | **Fold_change** | **-log10(*P*)** | **Abundance** | **Abundance %** | **Prevalence %** | **Change** | **Increase** |
| --- | --- | --- | --- | --- | --- | --- | --- | --- |
| Fusobacterium_7409 | Fusobacterium | -1.30 | 7.01 | 1921431 | 3.97% | 100.00 | 781164 | -1,140,267 |
| Neisseria_10019 | Neisseria | -3.32 | 23.88 | 1735113 | 2.75% | 99.24 | 173906 | -1,561,207 |
| Haemophilus_11091 | Haemophilus | -1.58 | 10.67 | 1199062 | 1.87% | 100.00 | 401269 | -797,793 |
| Veillonella_19390 | Veillonella | -0.88 | 5.09 | 817686 | 1.64% | 100.00 | 444007 | -373,679 |
| Gemella_3258 | Gemella | -1.08 | 5.78 | 714904 | 1.13% | 100.00 | 337850 | -377,054 |
| Porphyromonas_11896 | Porphyromonas | -3.04 | 18.95 | 491972 | 0.79% | 98.11 | 59970 | -432,002 |
| Prevotella_1969 | Prevotella | -1.13 | 3.67 | 346641 | 0.74% | 98.11 | 158588 | -188,053 |
| Leptotrichia_8995 | Leptotrichia | -2.31 | 10.74 | 324978 | 0.49% | 95.84 | 65668 | -259,310 |
| Leptotrichia_8876 | Leptotrichia | -2.50 | 4.75 | 215430 | 0.36% | 68.43 | 38202 | -177,228 |
| Neisseria_9962 | Neisseria | -2.91 | 23.19 | 158791 | 0.33% | 94.90 | 21191 | -137,600 |
| Prevotella_621 | Prevotella | -2.88 | 17.71 | 144656 | 0.29% | 93.57 | 19602 | -125,054 |
| Oribacterium_15965 | Oribacterium | -1.10 | 6.02 | 124840 | 0.28% | 99.05 | 58441 | -66,399 |
| Pasteurellaceae_11461 | Unknown | -2.14 | 10.61 | 123462 | 0.26% | 97.35 | 28037 | -95,425 |
| Capnocytophaga_2454 | Capnocytophaga | -3.24 | 15.96 | 113957 | 0.26% | 91.30 | 12083 | -101,874 |
| Capnocytophaga_509 | Capnocytophaga | -3.71 | 22.58 | 113027 | 0.22% | 93.57 | 8622 | -104,405 |
| Veillonella_19388 | Veillonella | -0.89 | 3.57 | 97522 | 0.22% | 99.43 | 52686 | -44,836 |
| Haemophilus_10797 | Haemophilus | -1.37 | 4.89 | 96360 | 0.19% | 97.54 | 37377 | -58,983 |
| Stomatobaculum_15638 | Stomatobaculum | -1.94 | 9.03 | 81904 | 0.18% | 90.93 | 21419 | -60,485 |
| Neisseria_9888 | Neisseria | -3.68 | 18.95 | 76462 | 0.17% | 80.91 | 5961 | -70,501 |

## Supplementary Table 6a. OTUs increased in asthmatics

| **OTU_ID** | **Genus** | **Phylum** | **Fold_change** | **-log10(*P*)** | **Abundance** | **Abundance %** | **Prevalence %** | **Change** | **Increase** |
| --- | --- | --- | --- | --- | --- | --- | --- | --- | --- |
| Neisseria_10019 | Neisseria | Proteobacteria | 0.96 | 1.45 | 1,371,169 | 4.74% | 99.44 | 2672809 | 1,301,640 |
| Rothia_13982 | Rothia | Actinobacteria | 0.78 | 1.46 | 65582 | 0.23% | 99.15 | 112474 | 46,892 |

## Supplementary Table 6b. OTUs decreased in asthmatics

| **OUT_ID** | **Genus** | **Fold_change** | **-log10(*P*)** | **Abundance** | **Abundance %** | **Prevalence %** | **Change** | **Increase** |
| --- | --- | --- | --- | --- | --- | --- | --- | --- |
| Actinomyces_13710 | Actinomyces | -0.63 | 2.01 | 1241550 | 4.29% | 100.00 | 805016 | -436,534 |
| Selenomonas_17559 | Selenomonas | -0.84 | 1.64 | 429036 | 1.48% | 100.00 | 239398 | -189,638 |
| Leptotrichia_8776 | Leptotrichia | -1.05 | 1.83 | 581845 | 2.01% | 99.72 | 281154 | -300,691 |
| Megasphaera_16215 | Megasphaera | -0.76 | 1.61 | 179586 | 0.62% | 99.72 | 105754 | -73,832 |
| Selenomonas_17440 | Selenomonas | -1.76 | 7.16 | 47875 | 0.17% | 99.44 | 14108 | -33,767 |
| Oribacterium_15113 | Oribacterium | -0.69 | 1.36 | 26997 | 0.09% | 97.18 | 16739 | -10,258 |
| Actinomyces_13062 | Actinomyces | -1.29 | 3.44 | 93637 | 0.32% | 95.77 | 38236 | -55,401 |
| Capnocytophaga_2454 | Capnocytophaga | -2.06 | 4.49 | 94930 | 0.33% | 93.24 | 22839 | -72,091 |
| Prevotella_879 | Prevotella | -0.99 | 1.45 | 56933 | 0.20% | 92.96 | 28662 | -28,271 |
| Streptococcus_4754 | Streptococcus | -0.55 | 1.45 | 6265 | 0.02% | 92.68 | 4275 | -1,990 |
| Selenomonas_17724 | Selenomonas | -1.28 | 2.59 | 24494 | 0.08% | 91.83 | 10101 | -14,393 |
| Candidate_division_TM7_12053 | Unknown | -0.93 | 1.48 | 39771 | 0.14% | 91.27 | 20826 | -18,945 |
| Streptococcus_6284 | Streptococcus | -0.74 | 1.83 | 6738 | 0.02% | 90.99 | 4041 | -2,697 |
| Prevotella_2754 | Prevotella | -1.61 | 2.32 | 113240 | 0.39% | 89.30 | 37057 | -76,183 |
| Actinomyces_13534 | Actinomyces | -0.64 | 1.52 | 6955 | 0.02% | 89.01 | 4456 | -2,499 |
| Ruminococcaceae_11808 | Unknown | -1.18 | 2.14 | 24385 | 0.08% | 88.73 | 10771 | -13,614 |
| Prevotella_2177 | Prevotella | -1.07 | 1.48 | 15489 | 0.05% | 85.07 | 7372 | -8,117 |
| Prevotella_2890 | Prevotella | -1.43 | 1.69 | 64968 | 0.22% | 83.38 | 24040 | -40,928 |
| Capnocytophaga_2417 | Capnocytophaga | -1.24 | 2.55 | 22882 | 0.08% | 82.82 | 9662 | -13,220 |
| Tannerella_312 | Tannerella | -1.72 | 2.69 | 29107 | 0.10% | 82.54 | 8860 | -20,247 |

## Supplementary Table 7. Analysis of *map* gene: frequencies and identities of *Streptococcus* spp.

Results are based on 475 samples, with 37,930,250 reads giving 14,898 *map* gene OTUs

| OUT ID | Identified Species | Abundance | Abundance % | Prevalence | Prevalence % | BLAST | | |  |
| --- | --- | --- | --- | --- | --- | --- | --- | --- | --- |
|  |  |  |  |  |  | **Identity (%)** | **E Value** | **Alignment** | **Tests Seen** |
| OUT10104 | *S. salivarius* | 601,651 | 1.59% | 474 | 99.79 | 99 | 0.00E+00 | 407/412 | 3 |
| OUT15936 | *S. salivarius* | 424,882 | 1.12% | 465 | 97.89 | 99 | 0.00E+00 | 408/412 | 3 |
| OUT13812 | *S. salivarius* | 169,574 | 0.45% | 256 | 53.89 | 98 | 0.00E+00 | 403/412 | 2 |
| OUT10307 | *S. salivarius* | 142,824 | 0.38% | 426 | 89.68 | 99 | 0.00E+00 | 410/412 | 4 |
| OUT24710 | *S. salivarius* | 96,238 | 0.25% | 457 | 96.21 | 99 | 0.00E+00 | 409/412 | 4 |
| OUT23481 | *S. salivarius* | 67,211 | 0.18% | 387 | 81.47 | 99 | 0.00E+00 | 408/412 | 3 |
| OUT21636 | *S. parasanguinis* | 47,040 | 0.12% | 373 | 78.53 | 98 | 0.00E+00 | 405/412 | 3 |
| OUT11843 | *S. salivarius* | 43,784 | 0.12% | 446 | 93.89 | 98 | 0.00E+00 | 403/412 | 3 |
| OUT13903 | *S. salivarius* | 43,697 | 0.12% | 366 | 77.05 | 99 | 0.00E+00 | 408/412 | 3 |
| OUT22893 | *S. salivarius* | 39,182 | 0.10% | 277 | 58.32 | 99 | 0.00E+00 | 406/412 | 2 |
| OUT10817 | *S. salivarius* | 38,512 | 0.10% | 364 | 76.63 | 99 | 0.00E+00 | 410/412 | 5 |
| OUT13837 | *S. parasanguinis* | 36,481 | 0.10% | 361 | 76 | 99 | 0.00E+00 | 411/412 | 3 |
| OUT10706 | *S. salivarius* | 35,934 | 0.09% | 189 | 39.79 | 98 | 0.00E+00 | 402/412 | 3 |
| OUT10102 | *S. salivarius* | 32,176 | 0.08% | 395 | 83.16 | 99 | 0.00E+00 | 411/412 | 3 |
| OUT9031 | *S. parasanguinis* | 28,211 | 0.07% | 143 | 30.11 | 94 | 1.00E-175 | 387/411 | 2 |
| OUT15267 | *S. parasanguinis* | 27,944 | 0.07% | 352 | 74.11 | 94 | 1.00E-175 | 387/411 | 3 |
| OUT12653 | *S. parasanguinis* | 27,913 | 0.07% | 349 | 73.47 | 95 | 0.00E+00 | 391/411 | 3 |
| OUT11394 | *S. oralis* | 27,602 | 0.07% | 184 | 38.74 | 85 | 1.00E-110 | 348/411 | 2 |
| OUT2846 | *S. salivarius* | 26,346 | 0.07% | 359 | 75.58 | 100 | 0.00E+00 | 412/412 | 3 |
| OUT124 | *S. parasanguinis* | 23,666 | 0.06% | 310 | 65.26 | 94 | 2.00E-172 | 385/411 | 3 |
| OUT10097 | *S. salivarius* | 23,030 | 0.06% | 451 | 94.95 | 99 | 0.00E+00 | 408/412 | 3 |
| OUT16404 | *S*. sp. I-G2 | 21,908 | 0.06% | 110 | 23.16 | 88 | 9.00E-132 | 359/408 | 2 |
| OUT2013* | *S. mitis* | 19,246 | 0.05% | 276 | 58.11 | 100 | 1.00E-180 | 348/348 | 4 |
| OUT10547 | *S. salivarius* | 18,369 | 0.05% | 316 | 66.53 | 99 | 0.00E+00 | 406/412 | 2 |
| OUT13462 | *S. parasanguinis* | 14,697 | 0.04% | 361 | 76 | 95 | 0.00E+00 | 392/411 | 3 |
| OUT12401 | *S. salivarius* | 9,055 | 0.02% | 377 | 79.37 | 99 | 0.00E+00 | 406/412 | 2 |
| OUT1213 | *S. thermophilus* | 6,355 | 0.02% | 265 | 55.79 | 98 | 0.00E+00 | 403/412 | 2 |
| OUT10860 | *S. parasanguinis* | 5,289 | 0.01% | 61 | 12.84 | 94 | 2.00E-177 | 388/411 | 2 |
| OUT12371 | *S. salivarius* | 3,705 | 0.01% | 354 | 74.53 | 99 | 0.00E+00 | 406/412 | 2 |
| OUT13964 | *S. salivarius* | 3,390 | 0.01% | 365 | 76.84 | 99 | 0.00E+00 | 406/412 | 2 |
| OUT23280 | *S. salivarius* | 3,128 | 0.01% | 254 | 53.47 | 99 | 0.00E+00 | 411/412 | 2 |
| OUT15822 | *S. salivarius* | 2,997 | 0.01% | 349 | 73.47 | 99 | 0.00E+00 | 411/412 | 2 |
| OUT14077 | *S. salivarius* | 2,635 | 0.01% | 347 | 73.05 | 99 | 0.00E+00 | 406/412 | 2 |

| Identity (%) | E Value | Alignment | Tests Seen |
| --- | --- | --- | --- |
| 94 | 1.00E-174 | 387/412 | 4 |

*OUT2013 potentially *S. pneumoniae* :

## Supplementary Table 8. *Streptococcus* spp. affected by smoking

| OTU ID | Identified Species | Abundance % | Prevalence % | DESeq analysis | |
| --- | --- | --- | --- | --- | --- |
|  |  |  |  | **Fold Change** | ***P* adjusted** |
| OTU10860 | *S. parasanguinis* | 0.01% | 12.84 | 5.2 | 1.75E-07 |
| OTU2013 | *S. mitis/pneumoniae* | 0.05% | 58.11 | 3.62 | 4.81E-09 |
| OTU24710 | *S. salivarius* | 0.25% | 96.21 | 3.03 | 5.59E-15 |
| OTU1213 | *S. thermophilus* | 0.02% | 55.79 | 2.53 | 7.38E-05 |
| OTU23280 | *S. salivarius* | 0.01% | 53.47 | 1.82 | 2.59E-04 |
| OTU12371 | *S. salivarius* | 0.01% | 74.53 | 1.53 | 9.00E-06 |
| OTU14077 | *S. salivarius* | 0.01% | 73.05 | 1.43 | 2.07E-05 |
| OTU13964 | *S. salivarius* | 0.01% | 76.84 | 1.34 | 1.00E-04 |
| OTU12401 | *S. salivarius* | 0.02% | 79.37 | -1.52 | 2.94E-04 |
| OTU10817 | *S. salivarius* | 0.10% | 76.63 | -1.92 | 3.56E-05 |
| OTU10706 | *S. salivarius* | 0.09% | 39.79 | -3.82 | 5.96E-08 |
| OTU11394 | *S. oralis* | 0.07% | 38.74 | -6.6 | 4.38E-17 |
| OTU16404 | *S*. sp. I-G2 | 0.06% | 23.16 | -8.44 | 2.91E-19 |
