# Supplementary material for: Airway microbial communities, smoking and asthma in a general population sample
Source: eBioMedicine. 2021 Aug 20;71:103538. doi: 10.1016/j.ebiom.2021.103538 (PMC8387768; doi:10.1016/j.ebiom.2021.103538)
Supplement: Supplementary file 2 [file mmc2.docx]

The supplementary file contains figures showing analysis schemes and population characteristics of the airway microbiome; and tables of subject characteristics, analyses of diversity determinants, Weighted Gene Correlation Network Analysis (WGCNA) components, and details of OTU abundance variation in smokers and asthmatics.
