# Supplementary material for: Airway microbial communities, smoking and asthma in a general population sample
Source: eBioMedicine. 2021 Aug 20;71:103538. doi: 10.1016/j.ebiom.2021.103538 (PMC8387768; doi:10.1016/j.ebiom.2021.103538)
Supplement: Supplementary file 3 [file mmc3.docx]

# Supplementary materials

Are contained within the accompanying file “Turek *et al.* Supplementary information.docx”
